# Spectrally PAINTing a Single Chain Polymeric Nanoparticle at Super-Resolution

**DOI:** 10.1021/jacs.2c11940

**Published:** 2022-12-14

**Authors:** Emmanouil Archontakis, Linlin Deng, Peter Zijlstra, Anja R. A. Palmans, Lorenzo Albertazzi

**Affiliations:** † Department of Biomedical Engineering and Institute for Complex Molecular Systems (ICMS), Eindhoven University of Technology, P.O. Box 513, 5600 MB Eindhoven, The Netherlands; ‡ Institute for Complex Molecular Systems (ICMS), Laboratory of Macromolecular and Organic Chemistry, Eindhoven University of Technology, P.O. Box 513, 5600 MB Eindhoven, The Netherlands; § Department of Applied Physics, Institute for Complex Molecular Systems, Eindhoven University of Technology, P.O. Box 513, 5600 MB Eindhoven, The Netherlands; ∥ Nanoscopy for Nanomedicine, Institute for Bioengineering of Catalonia, 08028 Barcelona, Spain

## Abstract

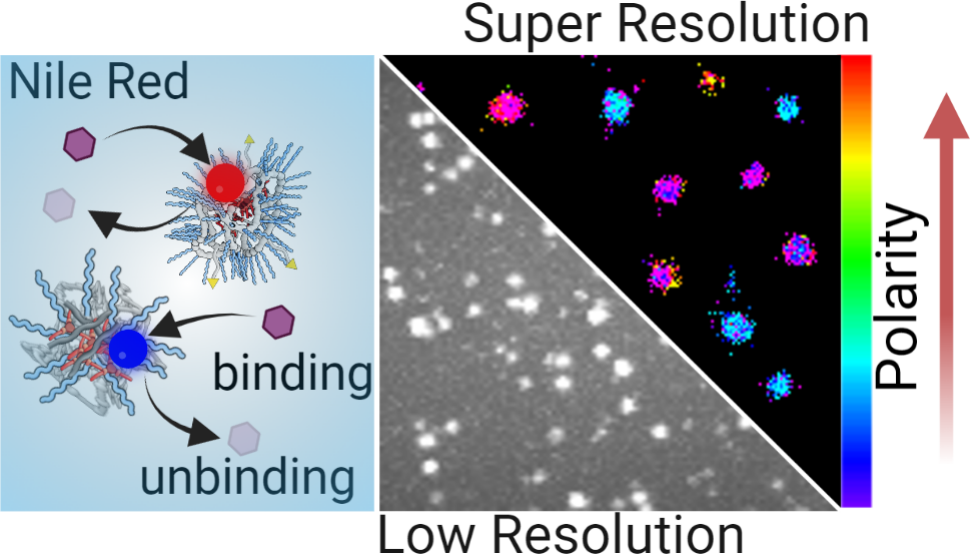

Folding a polymer chain into a well-defined single-chain polymeric nanoparticle (SCPN) is a fascinating approach to obtaining structured and functional nanoparticles. Like all polymeric materials, SCPNs are heterogeneous in their nature due to the polydispersity of their synthesis: the stochastic synthesis of polymer backbone length and stochastic functionalization with hydrophobic and hydrophilic pendant groups make structural diversity inevitable. Therefore, in a single batch of SCPNs, nanoparticles with different physicochemical properties are present, posing a great challenge to their characterization at a single-particle level. The development of techniques that can elucidate differences between SCPNs at a single-particle level is imperative to capture their potential applications in different fields such as catalysis and drug delivery. Here, a Nile Red based spectral point accumulation for imaging in nanoscale topography (NR-sPAINT) super-resolution fluorescence technique was implemented for the study of SCPNs at a single-particle level. This innovative method allowed us to (i) map the small-molecule binding rates on individual SCPNs and (ii) map the polarity of individual SCPNs for the first time. The SCPN designs used here have the same polymeric backbone but differ in the number of hydrophobic groups. The experimental results show notable interparticle differences in the binding rates within the same polymer design. Moreover, a marked polarity shift between the different designs is observed. Interestingly, interparticle polarity heterogeneity was unveiled, as well as an intraparticle diversity, information which has thus far remained hidden by ensemble techniques. The results indicate that the addition of hydrophobic pendant groups is vital to determine binding properties and induces single-particle polarity diversity. Overall, NR-sPAINT represents a powerful approach to quantifying the single-particle polarity of SCPNs and paves the way to relate the structural heterogeneity to functionality at the single-particle level. This provides an important step toward the aim of rationally designing SCPNs for the desired application.

## Introduction

Only a century after Staudinger postulated the concept of macromolecules, polymer chemists are able to control nearly every aspect of a polymer’s primary structure and architecture.^1^ In the 21st century, research efforts shifted toward the preparation of synthetic polymers that form confined, well-defined, three-dimensional structures, which express desired functions.^2^ This culminated in the development of the field of single chain polymeric nanoparticles (SCPNs), where single synthetic polymer chains collapse/fold into nanometer-sized particles, typically in a size range below 20 nm.^3−5^ In aqueous media, these soft nano-objects contain a hydrophobic interior that often arises from randomly distributed hydrophobic and/or supramolecular pendant groups attached to the polymer backbone. The compartmentalized, hydrophobic interior embedded in SCPNs has been explored for various applications such as catalysis,^6−9^ nanoreactors,^10,11^ drug carriers,^12^ and imaging agents.^13,14^

Alongside explorative studies highlighting the potential of SCPNs in a range of applications, fundamental studies have been conducted to elucidate the relationship between the polymer’s primary structure and the size, shape, and compactness of the formed SCPNs. Scattering techniques (X-ray and neutron scattering, dynamic and static light scattering) revealed that a balance between hydrophobic and hydrophilic groups is crucial to obtaining spherical, compact SCPNs.^15,16^ In addition, Overhauser dynamic nuclear polarization NMR showed that SCPNs with certain designs have structural and surface hydration properties reminiscent of those of enzymes.^17^ Moreover, fluorescence spectroscopy and confocal microscopy using the polarity-responsive dye Nile Red encapsulated inside the SCPNs elucidated how the stability of SCPNs in biological environments depended on the polymer’s primary structure.^18^

Whereas these ensemble techniques have provided valuable and detailed information on the structure of SCPNs with respect to their functionality, quantitative characterization of SCPNs at the single-particle level remains scarce.^19^ In contrast to enzymes, in which all polypeptide chains have a unique mass and primary structure, the random copolymers that afford SCPNs are intrinsically heterogeneous due to their molar mass dispersity and the random distribution of the functional groups in the copolymers.^20^ This is a key issue since these potential heterogeneities are expected to underline a diversity in the performance of SCPNs at the single-particle level.^21^ Therefore, a technique that can provide structural information beyond the ensemble would be of great interest in elucidating the relation between structural heterogeneities and functional performance for the rational design of SCPNs.

Super-resolution optical microscopy (nanoscopy) includes single-molecule techniques that surpass the diffraction limit, allowing for the visualization of nanomaterials with molecular specificity and sensitivity.^22−24^ Among them, points accumulation for imaging in nanoscale topography (PAINT) is a single-molecule fluorescence modality that can map the architecture of nanostructures with unprecedented lateral resolution of 5–25 nm.^25^ Fundamentally, PAINT is based on the detection of any freely diffusing fluorescent probe (e.g., DNA) that reversibly binds and unbinds to the target of interest, giving information about the number, distribution, and binding kinetics.^26^ To date, PAINT has been used as the gold standard for counting functional sites on nanoparticles,^27,28^ and has been extended to reveal peptides and lectin-sugar binding kinetics.^29,30^ Recently, PAINT has been updated to a spectrally resolved mode (sPAINT) using the fluorescent dye Nile Red which can change its emission color depending on the local polarity, enabling the simultaneous collection of the fluorescent molecule’s position and spectra.^31,32^ The possibility to perform spatially resolved spectroscopy opens many interesting new avenues especially when combined with dyes that can change emission wavelength in response to a specific cue (functional imaging). sPAINT has been applied for multicolor imaging of cells and for surface characterization.^33−35^ Recently, a similar spectrally resolved single-molecule approach was used to evaluate the functionality of antibody functionalized nanoparticles.^36^ However, characterization of synthetic polymers at a single particle level has not been reported. Note that the possibility of characterizing individual polymers in a batch is of great importance as it allows to disentangle the contribution of individual macromolecules in a very heterogeneous mixture.

In this work, the applicability of sPAINT combined with the solvatochromic dye Nile Red is presented, namely NR-sPAINT, with the aim to characterize the polarity of SCPNs on a single-particle basis and reveal the kinetics of binding and unbinding of Nile Red to different SCPNs.

NR-sPAINT provides a single Nile Red’s position and spectra near the binding site of the dye to a SCPN. Depending on the local polarity, Nile Red’s emission wavelength can either redshift (polar environment/less hydrophobic) or blueshift (less polar/more hydrophobic). By measuring the emission spectra of a large amount of Nile Red molecules that reversibly bind an individual SCPN, we can (i) detect a single Nile Red’s emission spectrum (which we refer to as a single molecule binding event) and from these (ii) extract a mean wavelength for each SCPN, which reflects their final single-particle mean polarity. Thus, the polarity of an individual SCPN can be extracted by calculating the mean value from all the Nile Red dyes that bind to this SCPN. This single-particle polarity quantification is an indication of the hydrophobicity of the nanoenvironment around SCPNs. Insight into the average hydrophobicity of the interior of SCPNs is important in view of catalytic applications, because the activity and selectivity of the catalytic sites, embedded inside the SCPN, is often related to the hydrophobicity of the interior.^11^

The SCPNs we use in this work are based on water-soluble, amphiphilic copolymers with randomly distributed hydrophobic/hydrophilic pendants, which form spontaneously when the copolymers are dissolved in water.^15^ To investigate how far the copolymer’s microstructure affects polarity differences of individual SCPNs, we use copolymer designs that comprise hydrophobic/hydrophilic grafts in different ratios. This results in SCPN populations that are more polar with a clear red shift of the emission spectra or, for the more hydrophobic designs, more apolar populations with a clear blue shift. The single-particle polarity results reveal structural heterogeneity within each batch of copolymers, denoting that structurally different SCPNs can exist in a single batch. Interestingly, an unprecedented intraparticle polarity diversity was also observed through Nile Red binding to a single SCPN. Our results provide insight into the heterogeneous structure of SCPNs at the single-particle level, information that remains hidden in SCPN ensemble techniques. Our approach allows for the first time to quantify the polarity of SCPNs at the single-particle level with single-molecule sensitivity and paves the way for the use of advanced super-resolution microscopy to guide the rational design of SCPNs.

## Results and Discussion

### NR-sPAINT Imaging of SCPNs

**Scheme 1:**
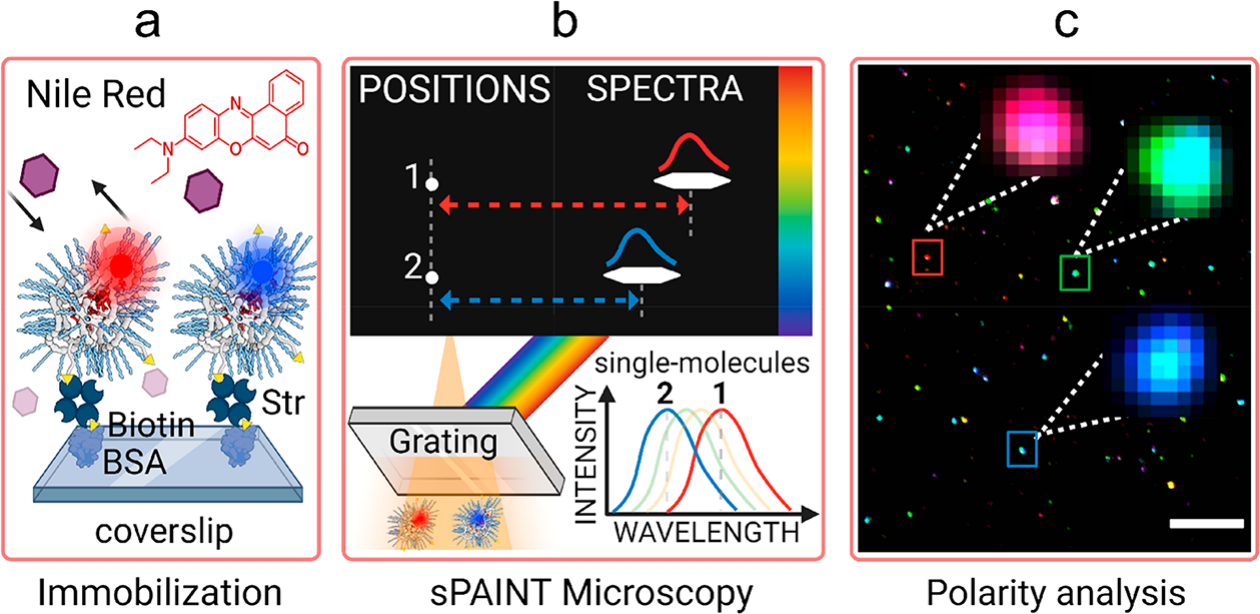
SCPN Immobilization Route and NR-sPAINT Microscopy to Measure the Mean Polarity of Individual SCPNs (a) Immobilization of the biotinylated SCPNs on a BSA/streptavidin (Str)-coated coverslip before imaging. The addition of Nile Red molecules in the solution generates transient hydrophobic interactions with SCPNs and subsequent release back into the water. (b) Principle of NR-sPAINT microscopy. During binding, the single Nile Red molecule is excited by the laser and the emitted light is transmitted through a diffraction grating in two different regions of the same detector generating a single positional spot (left) and a single stripe (right). The spot-stripe distance denotes the actual wavelength of the specific single Nile Red molecule. (c) NR-sPAINT visualization map of individual SCPNs. SCPNs appear with colors, representative of their mean polarity allowing for single-particle polarity analysis.

We first start with a brief overview on SCPN immobilization and the NR-sPAINT technique, and the workflow is presented in Scheme 1. All copolymers applied in this work are grafted with biotin and are dissolved into water to induce a hydrophobic collapse/folding, which results in the formation of SCPNs in solution. Then, bovine serum albumin (BSA)/streptavidin-coated coverslips were loaded with the biotinylated SCPNs (Scheme 1a). Subsequently, Nile Red was added to the solution which resulted in transient hydrophobic binding: (i) binding of a Nile Red molecule on a SCPN and (ii) escape of this Nile Red molecule from the SCPN (Scheme 1a). This procedure was iterated with a pool of Nile Red molecules present in the solution. A Nile Red molecule binding to the SCPN gives a fluorescence signal. The emission color of Nile Red depends on the polarity of the nanoenvironment that it senses; red depicts a more polar (or less hydrophobic) while blue depicts a more hydrophobic (or less polar) environment. NR-sPAINT imaging was accomplished by passing the detected Nile Red emission light through a transmission diffraction grating, which displays the position (single dot) and the unique spectrum (single stripe) of a single Nile Red molecule simultaneously (Scheme 1b). The dot-stripe distance defines the actual emission color of this Nile Red molecule. Finally, the superposition of the colors of all the single Nile Red molecules near individual SCPNs generate a spectrally resolved visualization map (Scheme 1c). Each zoomed square corresponds to an individual SCPN, and their colors depict their mean polarity and thus enable single-particle polarity analysis.

### Polymer Design and Synthesis

**Figure 1:**
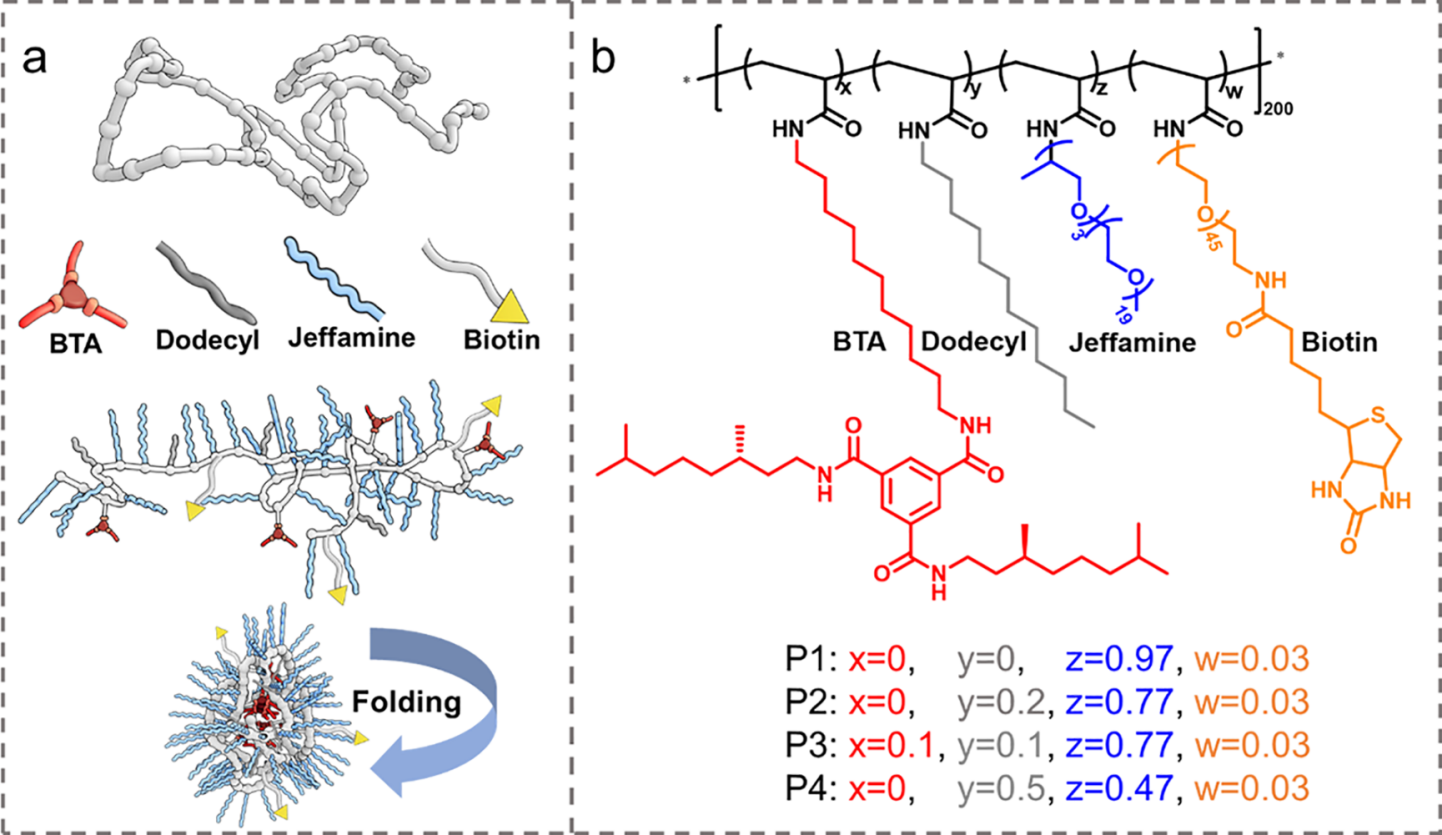
(a) Schematic illustration of the amphiphilic polymers that fold into SCPNs. (b) Chemical structure and composition of amphiphilic polymers **P1**–**P4** with hydrophobic (BTA, dodecyl) and hydrophilic (jeffamine, biotin) pendant groups.

Four amphiphilic polymers **P1**–**P4** were synthesized (Figure 1). In all cases, the same polymer precursor poly(pentafluorophenyl acrylate) with a degree of polymerization of 200 (pPFPA_200_) was used. Details of the synthesis and characterization of the copolymers are given in the Supporting Information (Figures S1–S13). All **P1**–**P4** comprise hydrophilic Jeffamine@1000 to ensure water-solubility. The more hydrophobic copolymers **P2**–**P4** comprise additionally the supramolecular motif benzene-1,3,5-tricarboxamide (BTA) and/or hydrophobic *n*-dodecylamine (Figure 1).^15^ The BTA pendants were selected because they aggregate via 3-fold hydrogen-bond formation, which induces a structured interior of the hydrophobic pocket of the SCPN. All copolymers also contain around 3% biotin (as determined by ^19^F-NMR), which can bind to a streptavidin functionalized surface with high affinity to ensure proper immobilization of the copolymers on the coverslip. **P1** is the most hydrophilic copolymer, without hydrophobic dodecyl or supramolecular BTA groups. **P2**–**P4** have increased hydrophobicity by introducing around 20% dodecyl groups to **P2**, 10% BTAs and 10% dodecyl groups to **P3**, and 50% dodecyl groups to **P4**. The 50% of hydrophobic pendants represents an upper limit in hydrophobic groups as higher contents typically result in multichain aggregation of the copolymers.^15^ With these pronounced differences in their primary structures, we cover a broad polarity spectrum of the copolymers, and hereby polarity differences between SCPNs that are expected to be amenable to NR-sPAINT characterization. It is important to note that the different types of pendant groups are randomly distributed in all copolymers, which ensures the formation of SCPNs when dissolved in water.^15^

First, we assessed if **P1**–**P4** form SCPNs when dissolved into water (Table 1). The hydrodynamic radius (*R*_H_) of nanoparticles formed from **P1**–**P4** was measured by dynamic light scattering (DLS). The results show *R*_H_ below 6.7 nm for all polymers (Figure S14). These values are in line with previous results.^15^ In addition, Nile Red was added to the solutions and the emission wavelength, λ_max,em_, was first measured using fluorescence spectroscopy. The results show λ_max,em_ of 664 nm for **P1**, and significantly lower values of around 650 nm for **P2**–**P3**, and the most blue-shifted of 644 nm for **P4** (Figure S15). These values indicate the presence of hydrophobic pockets in **P2**–**P4**, in line with previous results, and together with the *R*_H_ are a hallmark for SCPN formation.^18^ For **P1**, however, with a more red-shifted λ_max,em_ indicative for a less defined hydrophobic interior, it is likely that a more open, random coil-like conformation is adopted. To corroborate a structured interior when hydrogen-bonding groups are present, circular dichroism (CD) measurements were performed on **P3**. The chiral BTA grafts on **P3** trigger the folding of the polymer chain into compact conformations via 3-fold hydrogen-bond interactions, which is reflected by the presence of a Cotton effect.^4,17^Figure S16 of **P3** in water clearly shows a negative Cotton effect at 228 nm, indicating an *M* helical structure stabilized by 3-fold intramolecular hydrogen bonding. As **P1**–**P4** are decorated with biotin, which is known to bind streptavidin with high affinity, DLS was also applied for studying the interaction between biotinylated SCPNs and streptavidin. After the addition of streptavidin to **P1**–**P4** based nanoparticles in water, the size of nanoparticles increased slightly as illustrated in Figure S14 suggesting the successful binding between SCPN and streptavidin. Hence, all taken together, these results indicate that **P1**–**P4** form small, nanometer-sized particles in water and that the biotin pendants are capable of binding to streptavidin.

**Table 1:** Overview of the Chemical Composition of **P1**–**P4**, Number-Average Molecular Weight (*M*_n_), Molar Mass Dispersity (*Đ*_M_), and Hydrodynamic Radius (*R*_H_) of Nanoparticles Formed from **P1**–**P4**

	BTA^a^ (%)	dodecyl^a^ (%)	jeffamine^a^ (%)	biotin^a^ (%)	*M*_n,SEC_^b^ (kg mol ^–1^)	*Đ*_M_^b^	*R*_H_^c^ (nm)
P1	0	0	97	3	50.4	1.18	6.7
P2	0	20	77	3	46.8	1.21	5.7
P3	10	10	77	3	47.6	1.15	5.5
P4	0	50	47	3	44.8	1.10	4.6

a Based on the feed ratio which was confirmed via ^19^F NMR spectroscopy.

b Determined using SEC in DMF.

c Determined via DLS.

### NR-sPAINT on **P1**–**P4**-Based SCPNs Immobilized on a Surface

**Figure 2:**
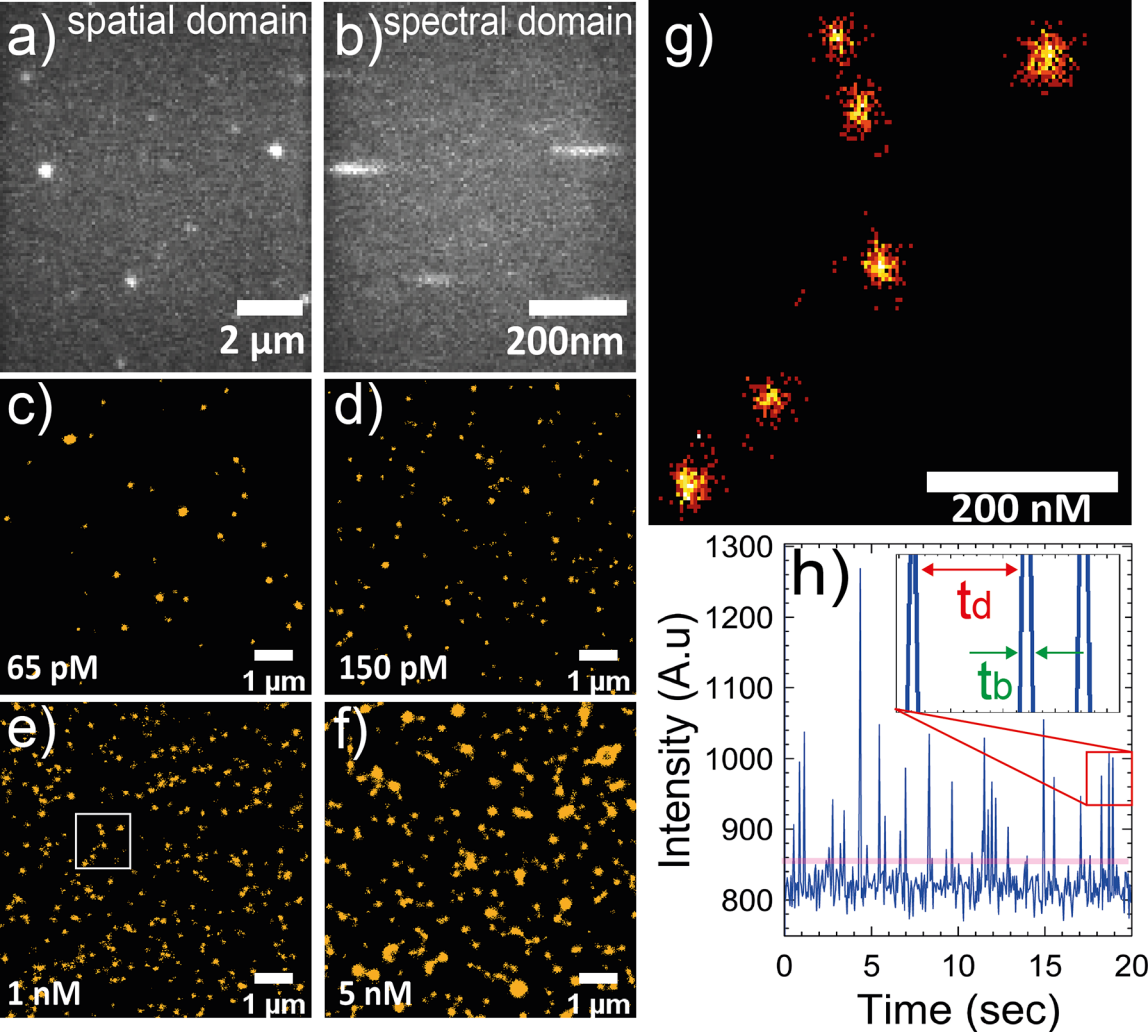
NR-sPAINT microscopy on SCPNs in water. (a) A single frame in a typical field of view (9 × 9) of a wide-field fluorescence microscope where each bright spot in the spatial domain encodes the position of a single Nile Red binding event around the SCPN. (b) The spectral domain of the same frame where each bright stripe corresponds to the emission wavelength of a specific Nile Red molecule detected in the spatial domain. (c–f) PAINT reconstruction maps of **P3**-based SCPNs, immobilized at concentrations of 65 pM, 150 pM, 1 nM, and 5 nM. (g) Density map of Nile Red binding events into the magnified inset at (e). Every single point represents a single Nile Red molecule event, and each cluster of events represents a SCPN. (h) Typical time trace of all events detected on an individual SCPN, which can be extracted from the spatial domain. The binding and unbinding of Nile Red molecules generate fluorescence bursts in time, characteristic of every single-molecule localization microscopy technique. (Inset) The duration of those bursts/events corresponds to the τ_b_-bright times and the distance between two bursts/events corresponds to the τ_d_-dark times. Nile Red imaging concentration: 5 nM. Total frames: 20 000.

With the procedure described in Scheme 1, Figure 2a,b illustrates the typical raw data (for a single frame) in NR-sPAINT microscopy. Details of the experimental setup and sample preparation are given in the Supporting Information. By using a diffractive optical grating before the detector, the emission light of an individual Nile Red molecule is split and directed into two paths: (i) the spatial domain representing the position of the excited Nile Red molecule (Figure 2a) and (ii) the spectral domain representing the emission spectrum of the Nile Red molecule (Figure 2b). For the former, the emission light is projected as a point spread function (bright spot), which encodes the position of the single Nile Red molecule (*X*, *Y*) and duration of its fluorescence (*T*) during binding to the SCPN, while the latter appears as an elongated point spread function (bright stripe) which encodes its wavelength (λ). This procedure iterates thousands of frames with thousands of Nile Red molecules which bind and unbind to SCPNs. The spot-stripe pixel distance can be calibrated and translated into the actual Nile Red emission wavelength, which is indicative of the local polarity of its environment (Figure S17–S18). The wavelength shifts that can be detected in this experimental setup are in the order of tens of nanometers, defined by the spectral precision of the setup (Figure S19). Considering the presence of the pool of Nile Red molecules in solution, specific binding events between the Nile Red and SCPNs are also an important factor in NR-sPAINT. To ensure that most Nile Red molecules bind specifically to the SCPN, control measurements were performed. Hence, a plane coverslip (absence of SCPN—control) was compared with a SCPN-functionalized coverslip (presence of SCPN—normal). The results showed that most of the clusters correspond to immobilized SCPNs (Figure S20). Additionally, the total number of Nile Red binding events is 4.2-fold higher in the presence of SCPNs compared to the one without, indicating that the specific Nile Red binding to the nanoparticles is dominant. The detection of SCPNs and the number of Nile Red events were evaluated using a MATLAB script previously described by us.^37^

As discussed, the biotinylated SCPNs were immobilized on a BSA/streptavidin-coated coverslip. Initially, the SCPN concentration was increased progressively to ensure that the particles can be immobilized successfully and in a controlled way, while the Nile Red concentration remained constant. The *X*, *Y*, *T* coordinates of each Nile Red obtained in thousands of frames can be merged to finally reconstruct a positional map of all Nile Red molecules together. This is illustrated in four representative reconstruction maps in Figure 2c–f obtained with different SCPN concentrations based on **P3** (65 pM, 150 pM, 1 nM, and 5 nM), all measured in water. By zooming into the selected area of interest in Figure 2e, we can see clusters. Each cluster in the field of view represents a SCPN. This is because when Nile Red molecules continue to bind and unbind the same SCPN, a cluster of points will form (Figure 2g). Each point in the cluster represents a Nile Red binding event. Varying the SCPN concentration leads to an increase of clusters in the field of view (Figure 2c–f), indicating that more SCPNs are being immobilized. As each SCPN cluster contains multiple bright spots which are driven by the reversible binding of the Nile Red molecules to the nanoparticle, the quantification of Nile Red molecules binding and unbinding to the SCPN at different time points can be depicted in Figure 2h. Practically, every time a Nile Red molecule binds to the SCPN, a burst of fluorescence will generate and last for a certain duration (τ_b_). Since many Nile Red molecules bind to the SCPN at different moments, there are many bursts at different times. When a Nile Red molecule unbinds the SCPN, the burst of fluorescence disappears and the waiting time for another burst to occur is defined as τ_d_; τ_b_ and τ_d_ refer to binding (or bright) time and unbinding (or dark) time, respectively, which are the two important metrics for kinetic quantification.^38^

### Quantification of Single-Particle Binding Kinetics Derived from Nile Red Binding Events

**Figure 3:**
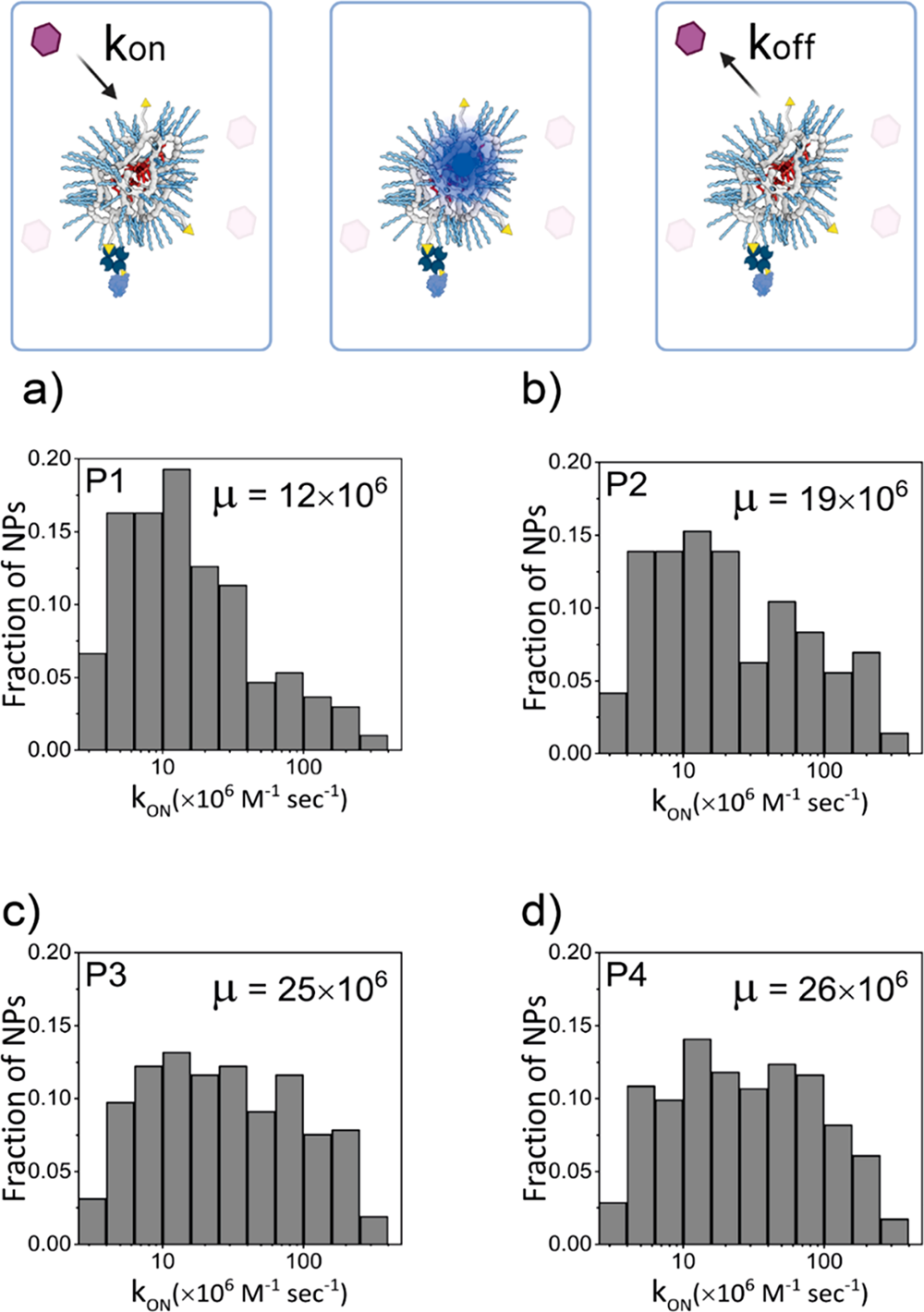
NR-sPAINT schematic representation and kinetic quantification histograms of Nile Red molecules (pink) which bind and unbind the SCPNs reversibly in the **P1**–**P4** series. This continuous and reversible procedure generates Nile Red binding events on each SCPN that can be quantified, to produce one single-particle association (*k*_on_) value, and thus statistical histograms for all SCPN values. (a–d) *k*_on_ histograms in **P1**–**P4** series. The addition of hydrophobic pendant groups does increase the fraction of SCPNs which exhibit faster *k*_on_ (μ corresponds to the median of the population). The width of each *k*_on_ histogram denotes a high interparticle dispersity within each design.

In PAINT, these two metrics are often expressed in kinetic rates (cartoon, Figure 3) as the unbinding rate (*k*_off_), which can directly be derived from the binding time from *k*_off_ = 1/τ_b_, and the binding rate (*k*_on_), which is also the reverse of dark time as shown in eq S1 in the SI.^39^ A single-particle quantification of the binding kinetic rates was further evaluated in order to address the accessibility of SCPNs by small molecules such as Nile Red. By extracting information of Nile Red unbinding and binding events per SCPN, it is possible to calculate the *k*_on_ and *k*_off_ for the single-particles in **P1**–**P4**, respectively (details in the Supporting Information).

The histograms in Figures 3a–d show the increasing tendency of the median *k*_on_ values in the series **P1**–**P4**, respectively. Specifically, in **P1** population (the most hydrophilic polymer), larger fractions of SCPNs have lower *k*_on_ (*k*_on_ < 30), while smaller fractions of SCPNs have almost double value (*k*_on_ > 30). In **P2**–**P3**–**P4** (the most hydrophobic polymers) a larger fraction of SCPNs that exhibits a higher association constant becomes more apparent with almost a 2- to 3-fold increase in the median of the total population (Figure S21). This confirms that indeed the functionalization of the backbone with higher amounts of hydrophobicity promotes the binding frequency in a large fraction of the total population. SCPNs with only hydrophilic groups, such as **P1**, likely form a more random-like coil structure, and thus exhibit more conformational flexibility as the hydrophobic collapse is less pronounced. This contrasts with **P2**–**P4** where the hydrophobic pendants collapse/fold the particles and induce a more compact, conformationally restrained structure. Hence, the conformational flexibility in **P1** makes it more difficult for the hydrophobic Nile Red molecules to access the hydrophobic domains of SCPNs, resulting in a fraction of SCPNs with decreased *k*_on_. For **P2**–**P4**, the increased number of hydrophobic grafts makes a fraction of SCPNs more static, which helps Nile Red access these nanoparticles in an easier manner due to the presence of more binding sites on the nanoparticle, resulting in an increased *k*_on._ More importantly, the single-particle heterogeneity in the association kinetic rates can be clearly observed in the **P1**–**P4** series. Although the median values of *k*_on_ do not show large shifts between different series, the increase of hydrophobic content in polymers causes a larger interparticle heterogeneity in association rates between individual SCPNs (1 order of magnitude), which is quantified for the first time exploiting the single molecule binding. In contrast, **P1**–**P4** show similar *k*_off_ trends regardless of the amount of hydrophobic groups (Figure S22). This can be explained by a limited sensitivity due to the lack of photostability of the Nile Red dye as well as to the limit of the exposure time of the camera, which does not allow for capturing shorter bright times, thus limiting the dynamic range of the detected *k*_off_ (Figure S23–S24).

### Quantification of Single-Particle Polarity Derived from Nile Red Binding Events

**Figure 4:**
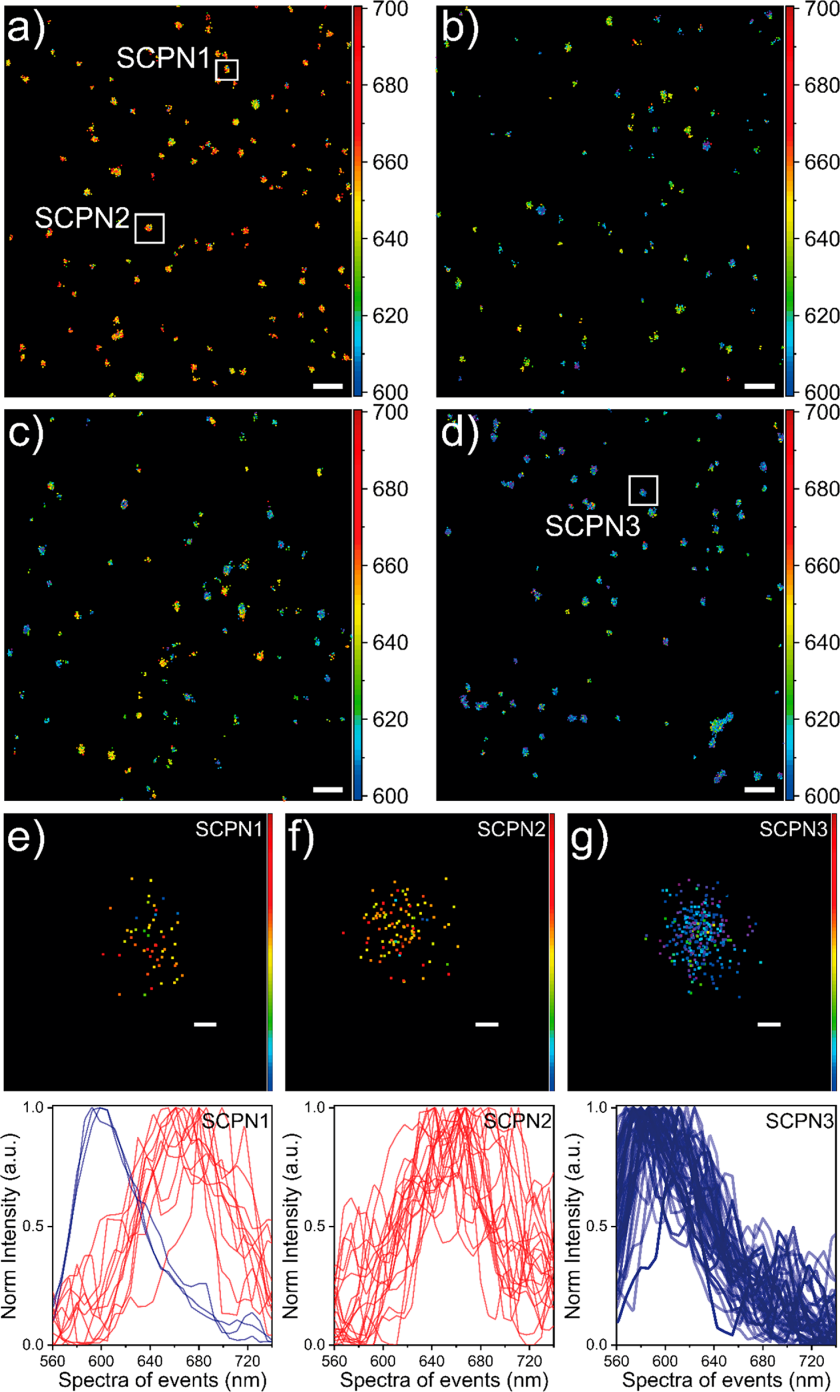
NR-sPAINT visualization. (a–d) Four representative spectrally resolved reconstruction maps in **P1**, **P2**, **P3**, and **P4**, respectively (scale bar: 500 nm). (e–g) The zoomed-in colorful clusters in a–d contain a finite amount of Nile Red binding events whose color (spectra) depends on the hydrophobicity of the local environment (scale bar: 25 nm). The more hydrophobic the more blue-shifted of the spectra of a single Nile Red molecule will be (blue points). The more hydrophilic the more red-shifted of the spectra of a single Nile Red molecule will be (red points). All the encoded Nile Red spectra per SCPN can be plotted and quantified by fitting them with a Gaussian function to extract the mean single-particle polarity.

Besides studying the binding kinetics via NR-sPAINT, each Nile Red molecule also senses a local polarity depending on the nanoenvironment to which it binds, and this is projected in the spectral domain of the detector. So, every Nile Red molecule carries not only positional and time information (*X*, *Y*, *T*) but also specific emission wavelength information (λ) that can be calculated by quantifying the spot-stripe distance. Then by calculating the mean peak wavelength of each single-Nile Red event which binds to an individual SCPN, a single-particle mean wavelength can be extracted. The value of the single-particle mean wavelength represents the average polarity of this specific SCPN. All Nile Red events have been assigned with a pseudocolor depending on the wavelength value; a red point represents a single Nile Red molecule that senses a hydrophilic environment, while a blue point represents a single Nile Red molecule that senses a more hydrophobic environment (Figure 4a–g). Different single-particle polarities between the designs are anticipated, which depend on the number of hydrophobic groups present on the polymer backbone: (i) a backbone free of hydrophobic groups will possibly form an interior of increased mean polarity (hydrophobicity decreases), while (ii) the addition of hydrophobic groups on the backbone will form an interior of decreased mean polarity (hydrophobicity increases). Finally, the polarity of hundreds of SCPNs can be calculated using NR-sPAINT. Specifically, the most hydrophilic **P1** contains a large fraction of SCPNs with red color. Nile Red molecules sense a polar environment in **P1** and shift their emission wavelengths to the red part of the spectrum (Figure 4a). By adding 20% dodecyl in **P2**, the reconstruction map shows a fraction of green and blue-shifted particles (Figure 4b); **P3**, which contains a mixture of dodecyl and 10% BTA pendants, behaves similar to **P2** (Figure 4c). The most hydrophobic polymer **P4** (containing 50% dodecyl group) exhibits a large fraction of SCPNs that show a blue color (Figure 4d and Figure 4g). Remarkably, the spectral reconstructions not only show the expected dispersity between the different copolymer microstructures, but also reveal an interparticle heterogeneity within the same polymer batch. This can be visualized in each polymer microstructure separately, which contains particles of different colors in the same batch (Figures 4a–d). For the first time, we can image the single-particle heterogeneity between batches as well as the diversity inside the same batch. Although the designs show similar trends in the ensemble/average (Figure S15), the underlying distributions are masked. NR-sPAINT can reveal the underlying distributions. Specifically, a fraction of intraparticle polarity diversity was observed, which shows that Nile Red molecules can feel different local environments during binding to the same SCPN, either more polar (less hydrophobic, thus red-shifted wavelengths) or less polar (more hydrophobic, thus blue-shifted wavelengths) (Figure 4e, SCPN1). The results indicate that local polarity around SCPN is very sensitive to the polymer’s microstructure and susceptible to large spectral shifts. NR-sPAINT is capable of quantifying SCPNs polarity heterogeneity in a single-particle fashion without any need of labeling, which may alter the polymer’s characteristics.

**Figure 5:**
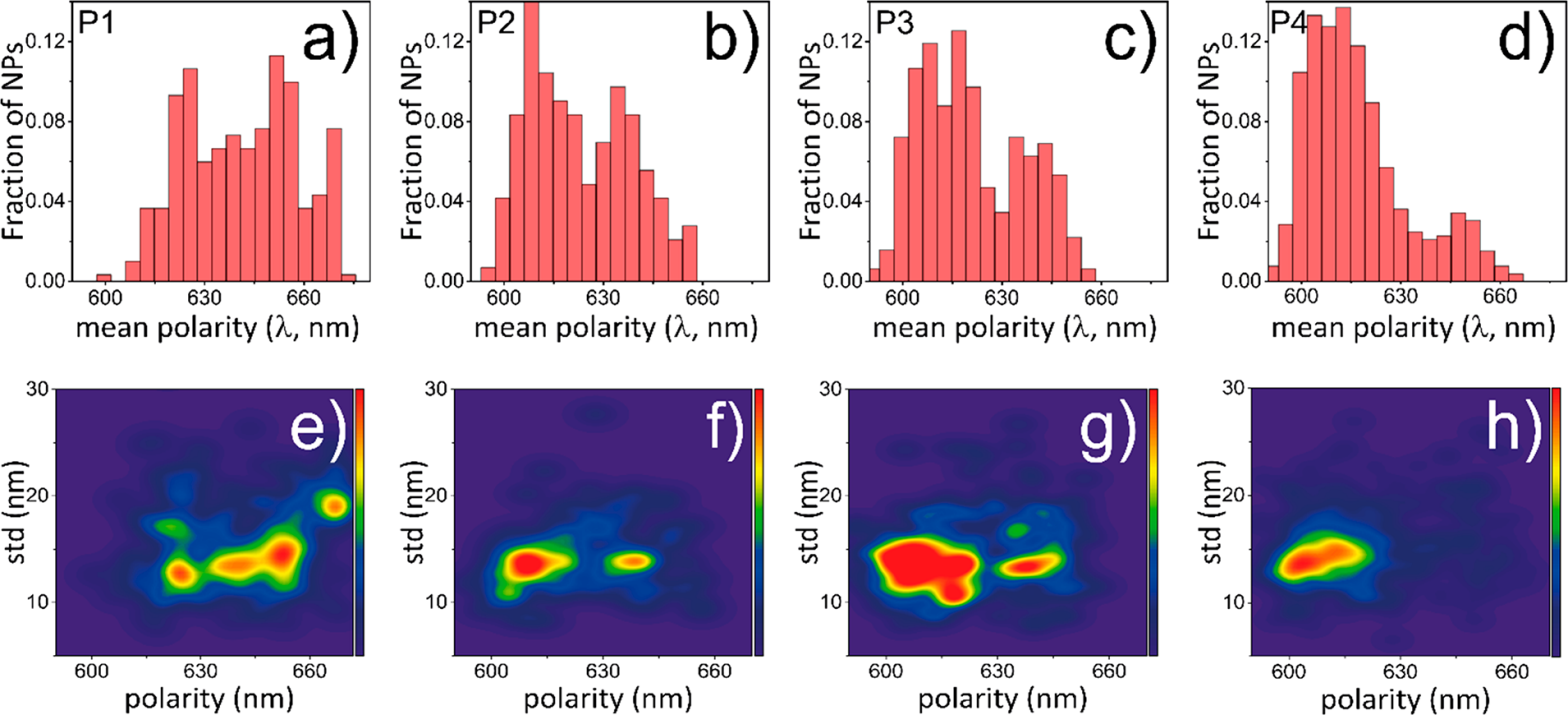
NR-sPAINT single-particle mean polarity quantification of **P1**–**P4**. (a–d) Frequency histograms of mean polarity values for single-particles in **P1**, **P2**, **P3**, and **P4**, respectively. (e–h) Mean polarity topography cloud using a probability density function. The color bar denotes the population density around a mean estimated polarity.

Next, we performed the spectral quantification of NR-sPAINT data based on all Nile Red binding events to different SCPNs (details of spectral quantification in the Supporting Information). The spectral quantification in **P1** showed a histogram with a mean SCPN polarity around 650 nm (Figure 5a). This value is close to the value in water (660 nm) and denotes the hydrophilic nature of **P1**. Going to more hydrophobic designs, such as **P2** and **P3**, the histogram of mean SCPN polarity is blue-shifted to around 625 nm (Figures 5b,c). The blue shift is most dominant for **P4**, resulting in a histogram with a mean SCPN polarity centered around 615 nm (Figure 5d). These values are similar to the emission values of Nile Red in organic solvents, such as methanol or acetone,^40^ suggesting that SCPNs provide a hydrophobic environment when Nile Red binds to these nanoparticles. Interestingly, there is a clear interparticle heterogeneity within the same batch of polymers for **P1**–**P4**. A fraction of SCPNs expresses a more polar interior (mean polarity > 630 nm) and a fraction expresses a less polar interior (mean polarity < 630 nm) existing in the same batch. The spectral shift (25 nm) is more than the spectral precision (10.7 nm) of the measurement (Figure S19). This implies that polarity variability emerges from diversity between single particles in the same batch.

Following the spectral quantification, a probability density function (PDF), which can estimate the relative likelihood of a continuous random mean polarity variable in our sample, was applied. Related to this, two single-particle metrics were calculated: (i) the mean polarity and (ii) the spectral standard deviation of the events to predict the underlining interparticle heterogeneity. This affords a 2D plot that provides a full picture of SCPN’s population heterogeneity. Starting from **P1**, a broad and dispersed mean polarity cloud with a dense SCPN polarity population centered around 650 nm is illustrated in Figure 5e. The most hydrophilic polymer **P1** is likely to adopt random coil conformations surrounded by water. When Nile Red molecules bind to domains which are exposed to the polar aqueous environment, the wavelength is red-shifted. Moreover, every time Nile Red binds to a SCPN formed by **P1**, it senses a different environment possibly due to the increased dynamic freedom of the **P1** particle that changes its structural conformation (random coil). This is expressed in the spectral standard deviation per particle, which reaches values close to 20 nm. Thus, a combination of polar and apolar nanoenvironments is exposed to Nile Red molecules. Upon the addition of hydrophobic groups in **P2**–**P3**, a mean blue-shift of the cloud and a less dispersed cloud compared to **P1** is observed, while still two populations of the cloud are observed: a fraction of single-particles that are more polar (650 nm) and a fraction that is less polar (620 nm) (Figure 5f,g). Interestingly, **P3** (Figure 5g) which contains supramolecular motif BTA shows a broader spectral standard deviation and polarity dispersity compared to **P2** (Figure 5f). This could be due to the arranging of BTAs via 3-fold hydrogen bonding into sparse conformations of polymers.^41^ This sparse conformation of nanoparticles induced by BTAs was reported in our previous work.^4,15^ In the case of **P4** with 50% dodecyl groups, a significant fraction of SCPNs is blue-shifted (centered around 615 nm), while the distribution becomes concentrated at shorter wavelengths and the spectral standard deviation at the single-particle level is smaller (Figure 5h). Based on the NR-sPAINT spectral visualization and quantification, the **P1** design which does not have any hydrophobic pendant group contains the most flexible and heterogeneous SCPNs while the addition of hydrophobic pendant groups such as in **P4** design expresses a more monodisperse distribution. This is corroborated by the fact that the more hydrophobic polymer has the higher chance for the SCPN to adopt a well-defined structure with a compact hydrophobic interior.

## Conclusions

In conclusion, NR-sPAINT was implemented to study how fast individual Nile Red molecules bind on polymeric nanoparticles and map their mean SCPN polarity using the dye’s solvatochromic nature. This method allows to get insights into the heterogeneities that arise from the stochastic nature of the polymer synthesis procedure. To enlighten this, NR-sPAINT takes advantage of the transient small-molecule binding to the SCPN, to capture the (i) spatiotemporal (position and time) and (ii) spectral information (polarity) of each binding event, simultaneously. Interparticle heterogeneities in structure were observed at different hierarchical levels of polymeric designs, for the first time.

First, the spatiotemporal quantification revealed an order of magnitude difference in small-molecule association rate between individual SCPNs within the same polymer family, indicating that structure variability remains high for random copolymer design. Additions of hydrophobic pendants to the polymers induce an increase in the fraction of SCPNs which exhibit higher association rates compared to more hydrophilic particles of the same design. The results suggest that the addition of hydrophobic pendant groups increases the frequency of binding events to some particles up to two times. This is also consistent with previous studies where different low affinity probes were used to map binding kinetics (DNA–DNA or glycan-lectin interactions).^42,30^ In a single batch of polymers, particles that contain bigger or smaller amounts of hydrophobic pendant groups exist and this can be probed using NR-sPAINT. Second, the spectroscopic quantification of each Nile Red molecule that binds on the SCPN allowed for single-particle polarity quantification. Intraparticle polarity heterogeneity close to 40 nm is observed. This may indicate the flexibility of conformation change within an individual SCPN, which was unmasked for the first time. Notably, interparticle polarity diversity among SCPNs from the same polymer family was also revealed, depicting that there are different populations of SCPNs: polar and more hydrophobic. The addition of hydrophobic pendant groups induces an almost 40 nm shift of the population to shorter wavelengths. Related to this, SCPNs based on more hydrophobic polymers tend to have a less dispersed mean polarity among individual nanoparticles, which depicts that the majority exhibits a confined nanoenvironment.

NR-sPAINT is a functional single-molecule imaging technique: one binding event at a time—one spectrum at a time. It encodes the local polarity of the polymer microstructure, by enabling quantitative interparticle and intraparticle analysis with molecular resolution, which otherwise would have been impossible with ensemble/average methods. NR-sPAINT can be used as a complementary tool and paves the way for the rational design of SCPNs or any other polymeric nanoparticle and further investigation of their structure and function relationship beyond the ensemble level.

## Supporting Information Available

Materials and Synthetic Procedures; Synthesis of **P1**–**P4**; Nanoparticle preparation and bulk characterization; Instruments; Sample preparation for NR-sPAINT measurements; Calibration of the instrument and spectral precision; Controls; Single molecule quantification; Supporting References (PDF)
