# Supplementary material for: Spectrally PAINTing a Single Chain Polymeric Nanoparticle at Super-Resolution
Source: J Am Chem Soc. 2022 Dec 14;144(51):23698–707. doi: 10.1021/jacs.2c11940 (PMC9801428; doi:10.1021/jacs.2c11940)
Supplement: Supplementary file 1 — ja2c11940_si_001.pdf [file ja2c11940_si_001.pdf]

Supporting Information

Spectrally PAINTing a Single Chain Polymeric Nanoparticle at Super- Resolution

Emmanouil Archontakis[1┼], Linlin Deng[2┼], Peter Zijlstra[3*], Anja R. A. Palmans[2*], Lorenzo Albertazzi[1,4*]

[1] Department of Biomedical Engineering and Institute for Complex Molecular Systems (ICMS), Eindhoven University of Technology, P.O. Box 513, 5600 MB Eindhoven, The Netherlands

[2] Institute for Complex Molecular Systems (ICMS), Laboratory of Macromolecular and Organic Chemistry, Eindhoven University of Technology, P.O. Box 513,5600 MB Eindhoven, The Netherlands

[3] Department of Applied Physics, Institute for Complex Molecular Systems, Eindhoven University of Technology, P.O. Box 513,5600 MB, Eindhoven, The Netherlands

[4] Nanoscopy for Nanomedicine, Institute for Bioengineering of Catalonia, Barcelona, Spain

┼ These authors have contributed equally

Table of Contents

1. Materials & Synthetic Procedures 2
2. Synthesis of P1-P4 3
3. Nanoparticle preparation and bulk characterization 11
4. Instruments 14
5. Sample preparation for NR-sPAINT measurements 14
6. Calibration of the instrument & spectral precision 16
7. Controls 19
8. Single molecule quantification 20
9. References 23

1. Materials & Methods

Poly(ethylene glycol) 2-aminoethyl ether biotin (average M_n = 2,300 g/mol) was purchased from Sigma Aldrich. Jeffamine®M-1000 polyetheramine was obtained from Huntsman. AIBN was recrystallized from methanol prior to use. Other reagents for polymer synthesis were purchased from Tokyo Chemical Industry (TCI). Deuterated solvents were purchased from Cambridge Isotopes Laboratories and other solvents were obtained from Biosolve. Dry solvents were used from an MBRAUN Solvent Purification System (MB-SPS). BTA amine[1] was synthesized according to a published procedure. Dialysis membranes were purchased from Spectrum Laboratories, with a molecular weight cut off of 6-8 kDa. Biotinylated bovine serum albumin and streptavidin were purchased from Thermo Fisher Scientific Massachusetts, US.
All ^{1}H NMR spectra were obtained using either on a Varian Mercury Vx 400 MHz or a Varian 400MR 400 MHz and recorded in $CDCl_3$ (internal reference 7.26 ppm). The ^{1}H-NMR chemical shifts are reported in ppm downfield from tetramethylsilane (TMS). CD spectroscopy experiments were performed on a JASCO J-815 spectropolarimeter, at 20 °C using a JASCO CTU100 Circulating Thermoset Unit. The optical path width of the cells was 10 mm. Nile Red fluorescence measurements with polymers in water were performed on a Varioskan Lux Multimode Microplate Reader (ThermoFisher Scientific). DMF-SEC measurements were carried out in PL-GPC-50 plus from Polymer Laboratories (Varian Inc. Company) equipped with a refractive index detector and elution solvent DMF containing 10 mM LiBr at 50 °C (flow rate: 1 mL min^{-1}) on a Shodex GPC-KD-804 column (exclusion limit = 400 kDa.; 0.8 cm i.d. × 300 mm) or on a Shodex GPC-KD-805 column (exclusion limit = 5000 kDa.; 0.8 cm i.d. × 300 mm). The columns were calibrated with poly(ethylene oxide) (PEO) samples (Polymer Laboratories). Dynamic light scattering (DLS) measurements were performed on a Malvern mV Zetasizer equipped with an 830 nm laser and a scattering angle of 90°. Data export using Zetasizer software provided by Malvern Instruments to give the correlation functions and the distributions of the hydrodynamic radius.

2. Synthesis of P1-P4 SCPNs

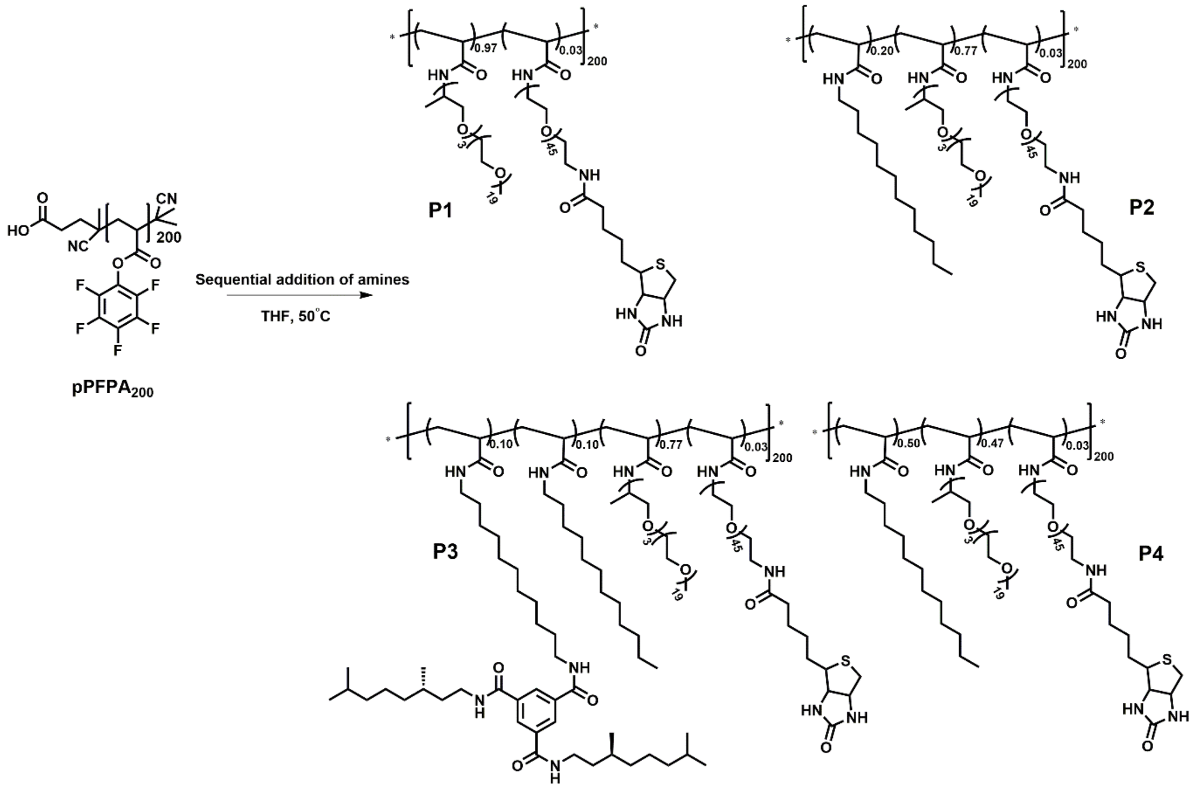

Polymer precursor pPFPA$_{200}$ was prepared following our previous work.[1,2]

Polymer precursor pPFPA$_{200}$ (200 mg, 4 eq) and biotinylated PEG amine (57.6 mg, 24 eq) were added to the Schlenk tube in dry THF (10 mL). The reaction mixture was degassed under argon for 30 minutes. After that, the reaction was stirred at 50 °C for 2 h under argon, and the mixture was taken for ^{19}F-NMR spectroscopy to determine the degree of functionalization. Then the mixture was divided equally into four batches. For batch one, dry Jeffamine M-1000 (405 mg, 388 eq) was added to the solution and stirred at 50 °C overnight to prepare **P1**. For batch two, *n*-dodecylamine (7.7 mg, 40 eq) was added in dry THF (3 mL) and after 2 h, the mixture was taken for ^{19}F-NMR spectroscopy to check the degree of functionalization for dodecylamine. Finally, dry Jeffamine M-1000 (322 mg, 308 eq) was added to the solution and stirred at 50 °C overnight to prepare **P2**. For batch three, BTA-amine (13.7 mg, 20 eq) in dry THF (3 mL) was added and stirred for 2 h at 50 °C. The sample was then taken for ^{19}F-NMR spectroscopy to check the degree of functionalization for BTA. *n*-Dodecylamine (3.9 mg, 20 eq) in dry THF (3 mL) was then added to the reaction mixture at 50 °C. The degree of functionalization for

dodecylamine was monitored via ^{19}F-NMR spectroscopy. Finally, dry Jeffamine M-1000 (322 mg, 308 eq) was added to the solution and stirred at 50 °C overnight to prepare **P3**. For batch four, dodecylamine (19.3 mg, 100 eq) in dry THF (3 mL) was added to the reaction mixture. After 2 h, the sample was taken for ^{19}F-NMR spectroscopy. Finally, dry Jeffamine M-1000 (196 mg, 188 eq) was added to the solution and stirred at 50 °C overnight to prepare **P4**. To confirm the end of post-functionalization, the reaction mixture of each batch was again taken for ^{19}F-NMR spectroscopy. The purification of polymer was performed via dialysis in THF for two days, and then in methanol for two days to remove all the released pentafluorophenol and free Jeffamine.

The ratios of the different grafts were determined using ^{19}F-NMR and corroborated by ^{1}H-NMR. For ^{19}F-NMR the integrated area of the released pentafluorophenol was compared to that of the pendant pentafluoromethyl groups. For ^{1}H-NMR, signals that were unique for a specific type of hydrogen were integrated and compared. For example, we can see that each compound, BTA, biotin, Jeffamine and dodecyl in Figure S13, has a unique peak in their ^{1}H-NMR spectra. For example, the protons of methyl group in Jeffamine labeled with orange dots are located at ~1.13 ppm; this peak is not overlapping with the peaks of biotin and dodecyl. We assume that the sum of functional groups grafted to corresponding polymers account for 100%. We used these peaks in the ^{1}H-NMR spectra of **P1-P4** to estimate the incorporation ratios. For polymers **P1**, **P2** and **P4**, which contain only biotin, Jeffamine and/or dodecyl, the grafted dodecyl can be calculated by comparing the peak integration at ~1.13 ppm (Jeffamine), ~1.26 ppm (dodecyl) and ~4.5 ppm (biotin). For **P1**, there is no peak around 1.26 ppm (Figure S2), indicating 0% dodecyl in **P1**. By comparing the integration between ~1.13 ppm (Jeffamine) and ~4.5 ppm (biotin), the calculated amount of biotin in **P1** is around 5.6%. Considering the likely error in the integration of such small peaks in the ^{1}H-NMR spectra, we believe that 5.6% is in good agreement with the 3% conversion obtained by ^{19}F-NMR. Similarly, for **P2**, the amount of dodecyl and biotin in the polymer is around 22.9% and 5.7% (Figure S5). For **P4**, the amount of dodecyl and biotin in polymer is around 51.3% and 5.6% (Figure S11). For **P3** (Figure S8) which contains BTA, as protons (16 H) signal of BTA also contributes to the peak of dodecyl around ~1.26 ppm, the percentage of BTA and dodecyl in total can be roughly calculated around 24.6%, while BTA can be calculated from ~8.34 ppm around 13%, the dodecyl amount is thus calculated around 11.6%.

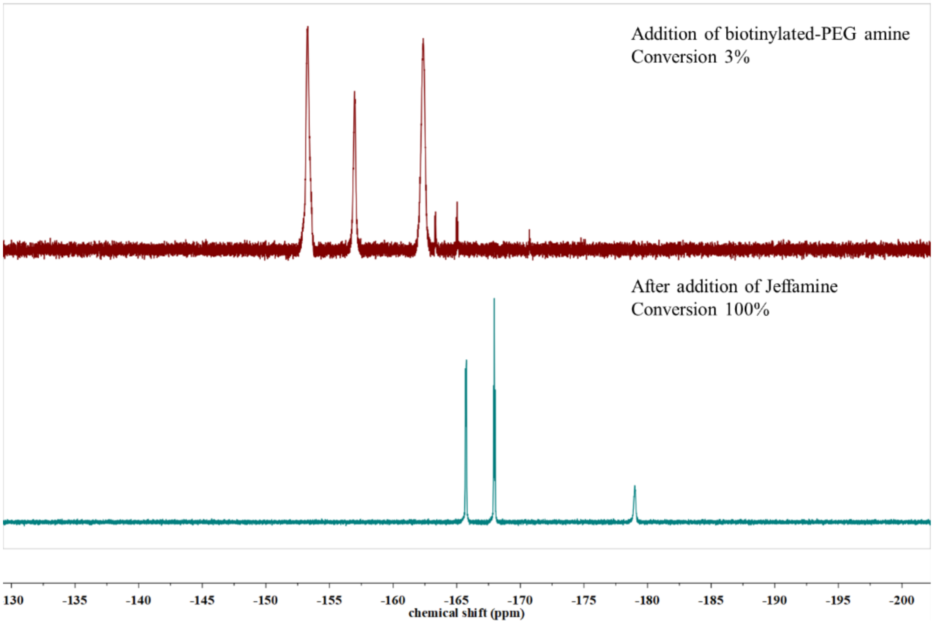

Figure S1: ^{19}F-NMR spectra in $CDCl_3$ of sequential functionalization of $pPFPA_{200}$ into **P1.**

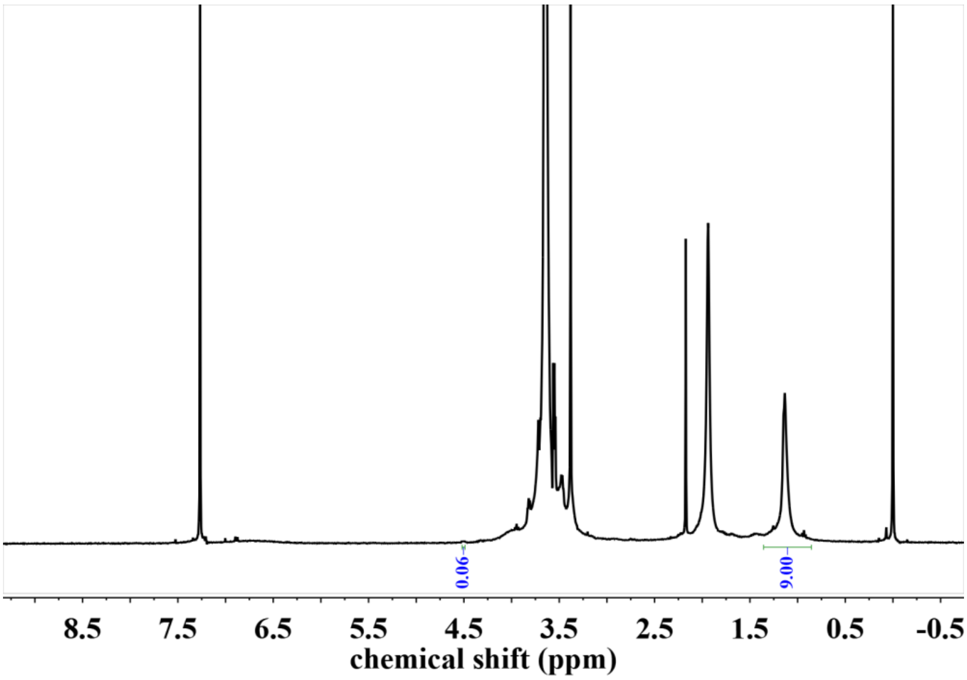

Figure S2: The ^{1}H-NMR spectrum in $CDCl_3$ of **P1**. The integrals of the relevant peaks for calculating the ratio of the different pendants are indicated.

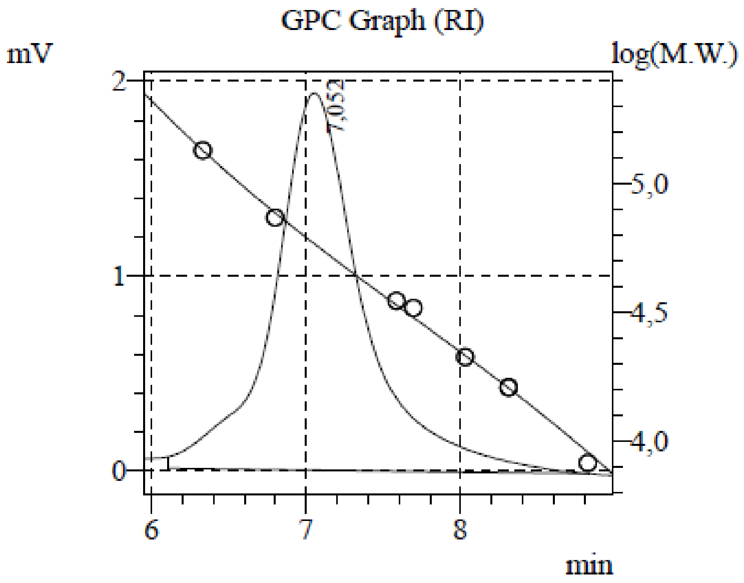

Figure S3: SEC of **P1** in DMF. The molecular weight characteristics of this polymer are M_n = 50.4 kg/mol, $Đ_M$ = 1.18.

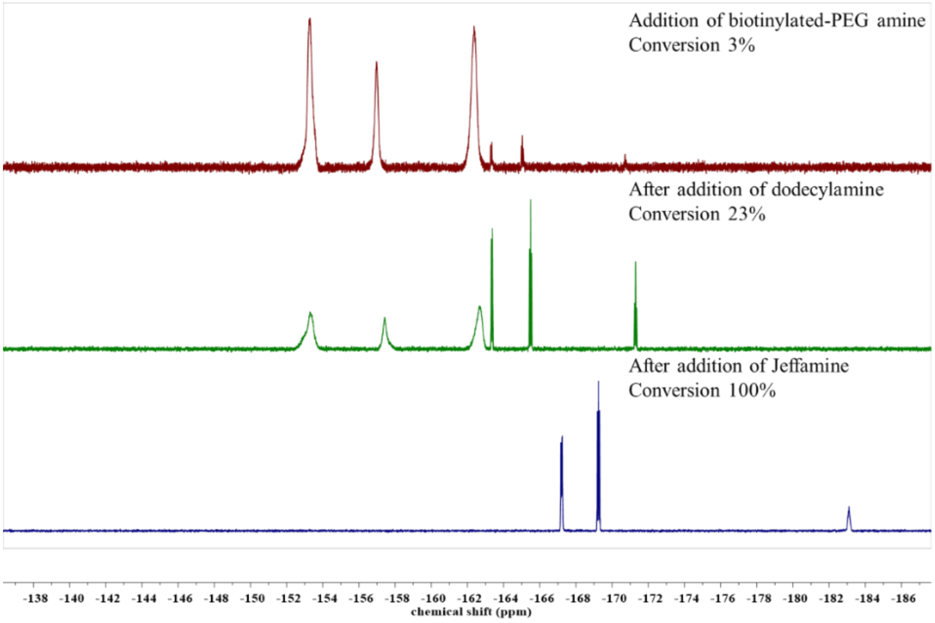

Figure S4: ^{19}F-NMR spectra in $CDCl_3$ of the sequential post-polymerization of $pPFPA_{200}$ into **P2.**

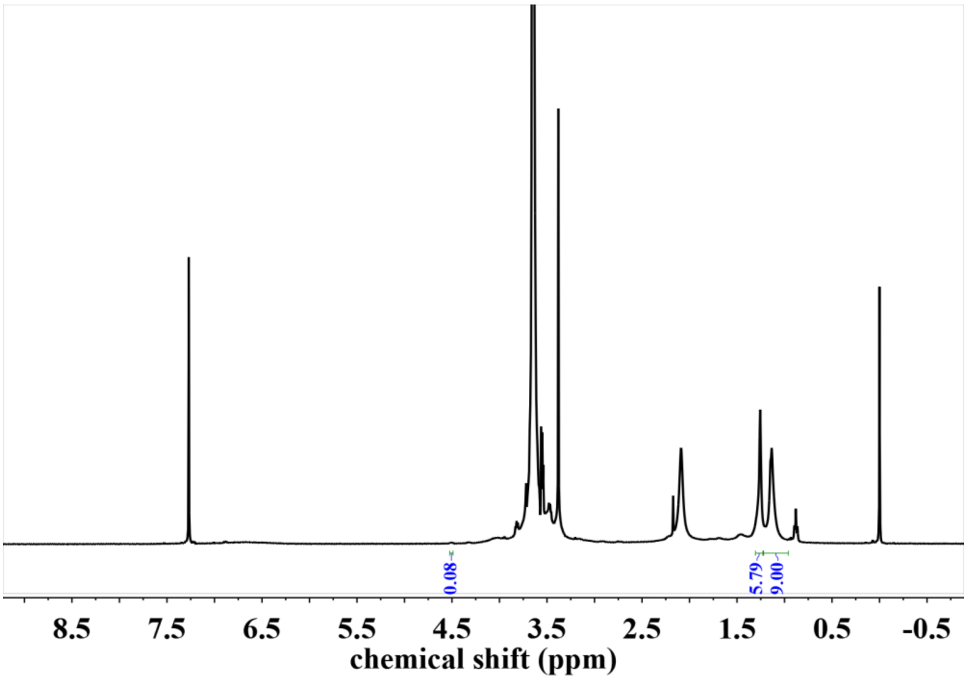

Figure S5: The ^{1}H-NMR spectrum of **P2** in $CDCl_3$. The integrals of the relevant peaks for calculating the ratio of the different pendants are indicated.

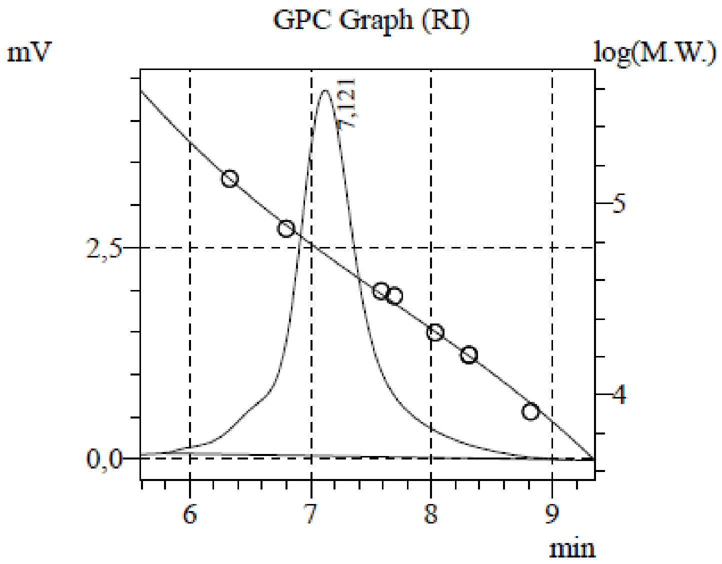

Figure S6: SEC of **P2** in DMF. The molecular weight characteristics of this polymer are M_n = 46.8 kg/mol, $Đ_M$ = 1.21.

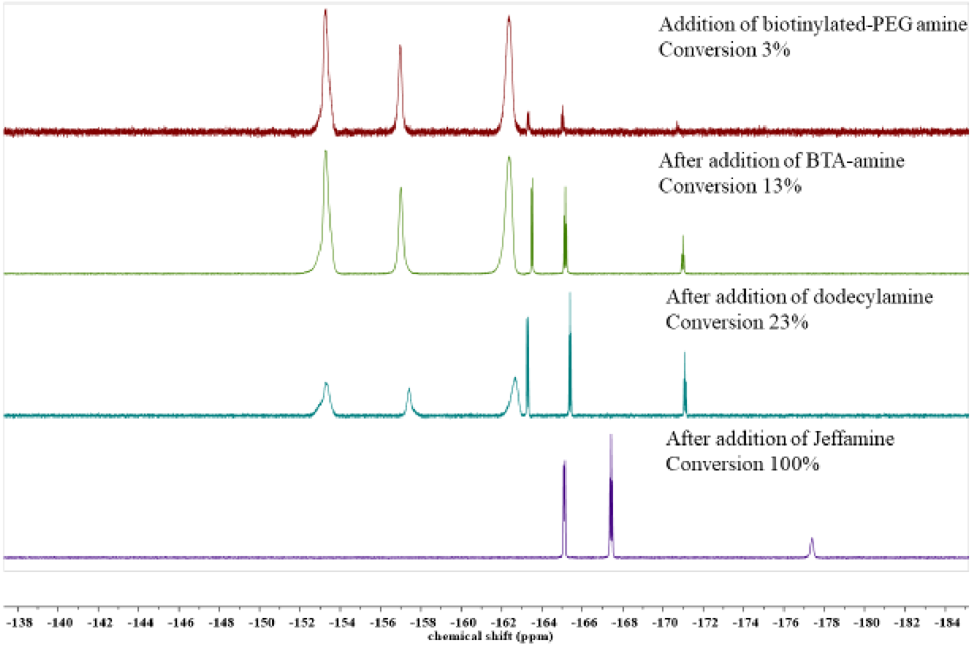

Figure S7: ^{19}F-NMR spectra in $CDCl_3$ of the sequential post-polymerization of pPFPA$_{200}$ into **P3.**

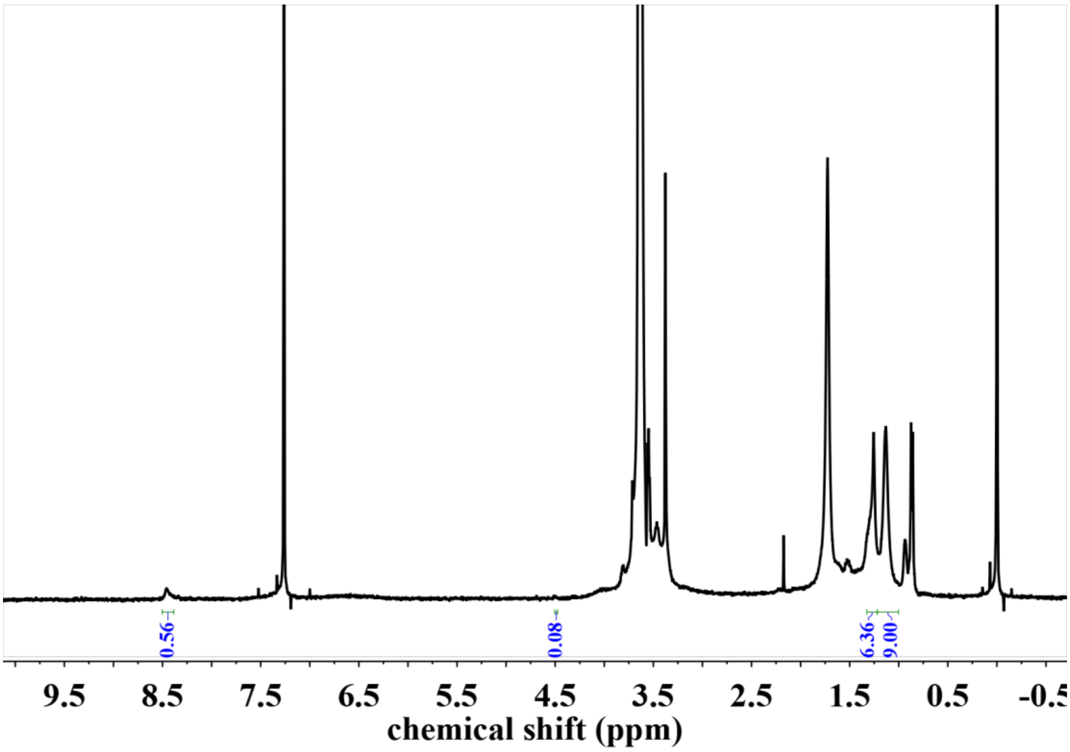

Figure S8: The ^{1}H-NMR spectrum of **P3** in $CDCl_3$. The integrals of the relevant peaks for calculating the ratio of the different pendants are indicated.

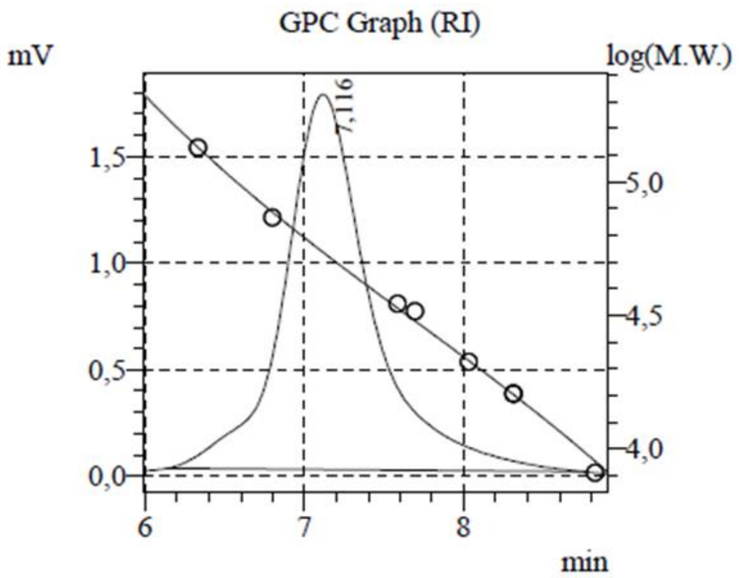

Figure S9: SEC of **P3** in DMF. The molecular weight characteristics of this polymer are M_n = 47.6 kg/mol, $Đ_M$ = 1.15.

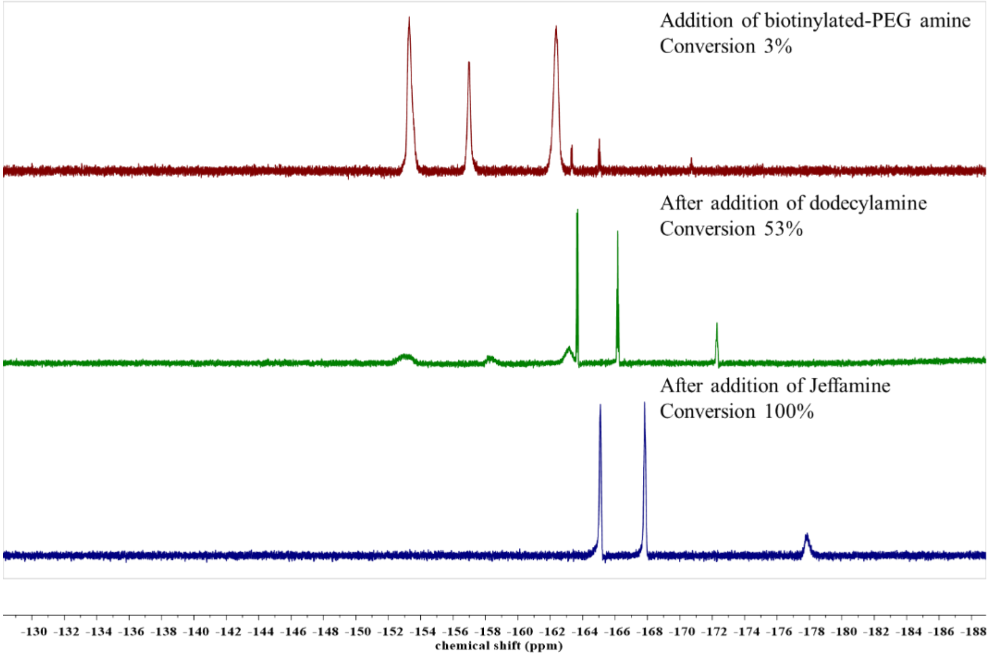

Figure S10: ^{19}F-NMR spectra in $CDCl_3$ of the sequential functionalization of $pPFPA_{200}$ into **P4.**

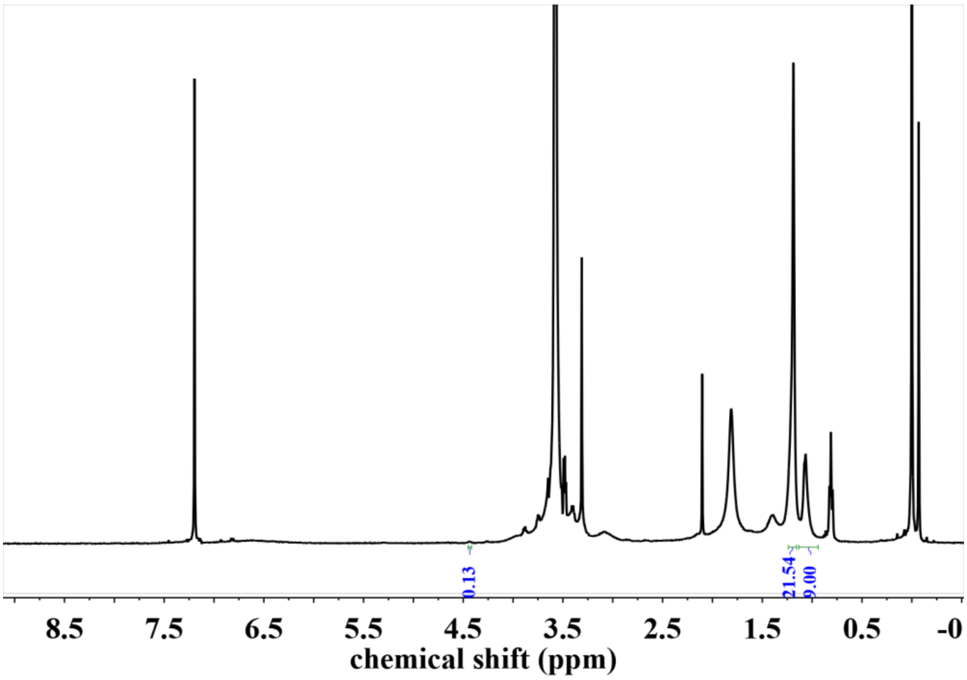

Figure S11: The ^{1}H-NMR spectrum of **P4** in $CDCl_3$**.** The integrals of the relevant peaks for calculating the ratio of the different pendants are indicated.

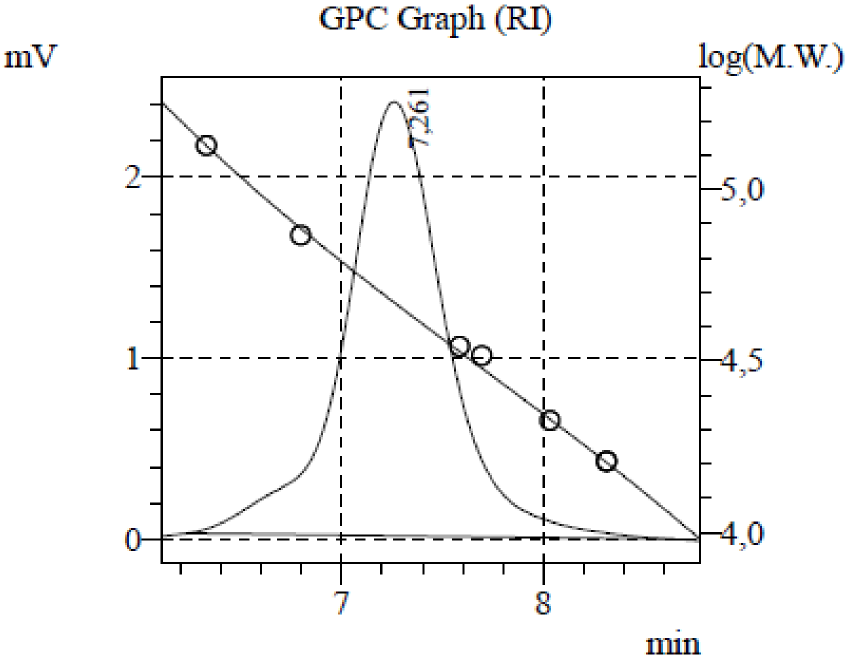

Figure S12: SEC of **P4** in DMF. The molecular weight characteristics of this polymer are M_n = 44.8 kg/mol, $Đ_M$ = 1.10.

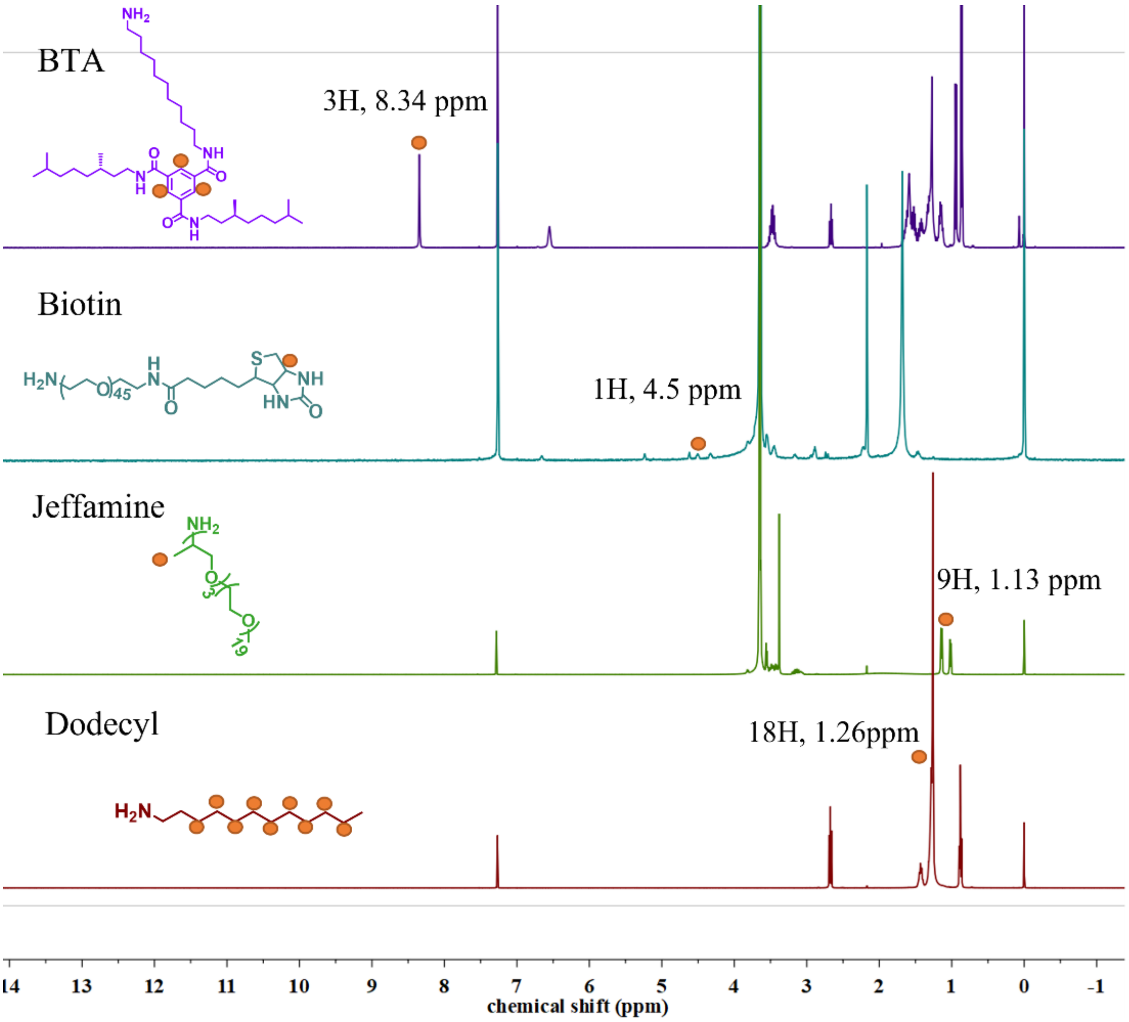

Figure S13: ^{1}H-NMR of BTA, Biotin, Jeffamine and dodecyl. Unique signals of each compound that do not overlap with other compounds are indicated with the orange circle.

3. Nanoparticle preparation and bulk characterization

Polymers **P1−P4** were directly dissolved in Milli-Q water at a concentration of 4.2 µM. The mixture was vortexed and then heated at 80 °C for 1 hour. The solution was cooled to room temperature and was equilibrated overnight at room temperature prior to measurements. For ensemble fluorescence measurements all polymer designs in water (4.2 µM) were mixed individually with Nile Red (1 µM) into a Microplate Reader in a total volume of 100 µL.

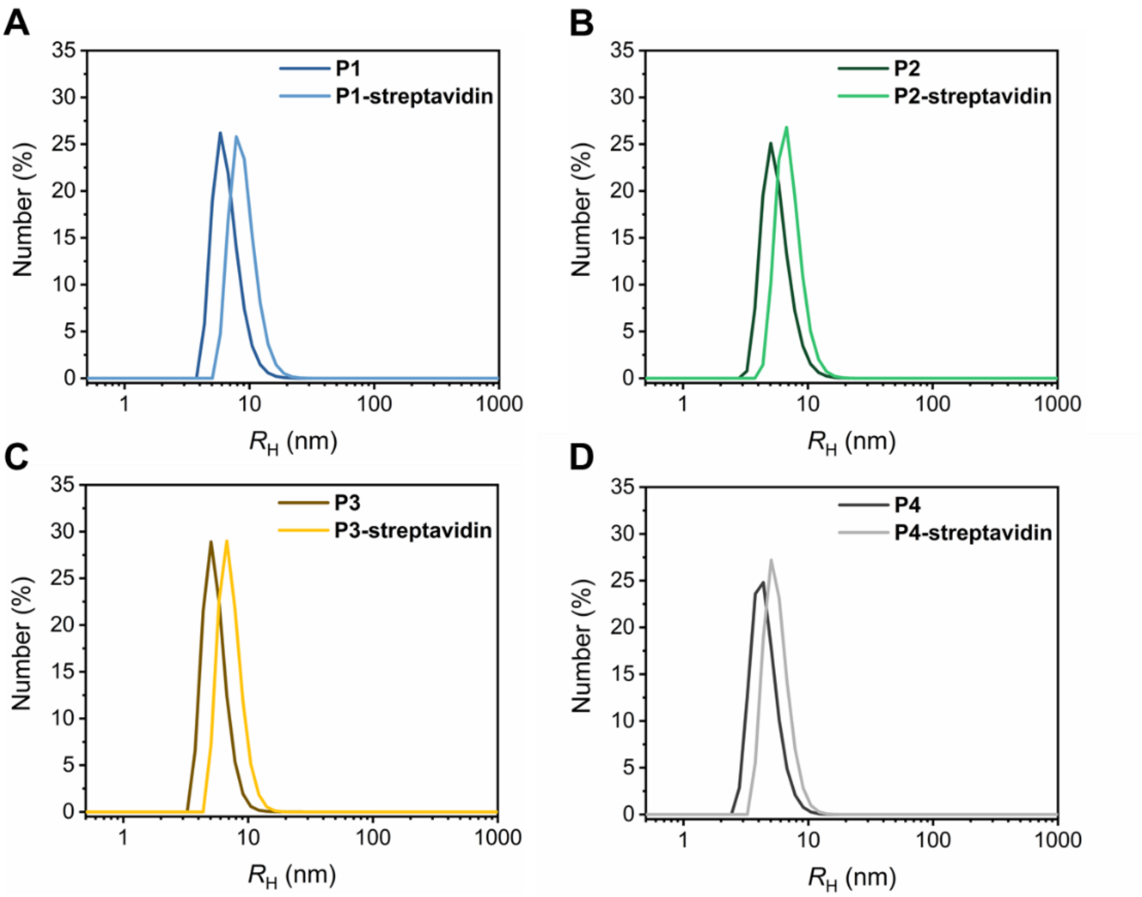

Figure S14: DLS measurements of (A) **P1** (R_H = 6.7 nm), (B) **P2** (R_H = 5.7 nm), (C) **P3** (R_H = 5.5 nm), (D) **P4** (R_H = 4.6 nm) (concentration = 4.2 μM, T = 20 °C) in water before and after mixing with streptavidin (0.03 mg mL^{-1}).

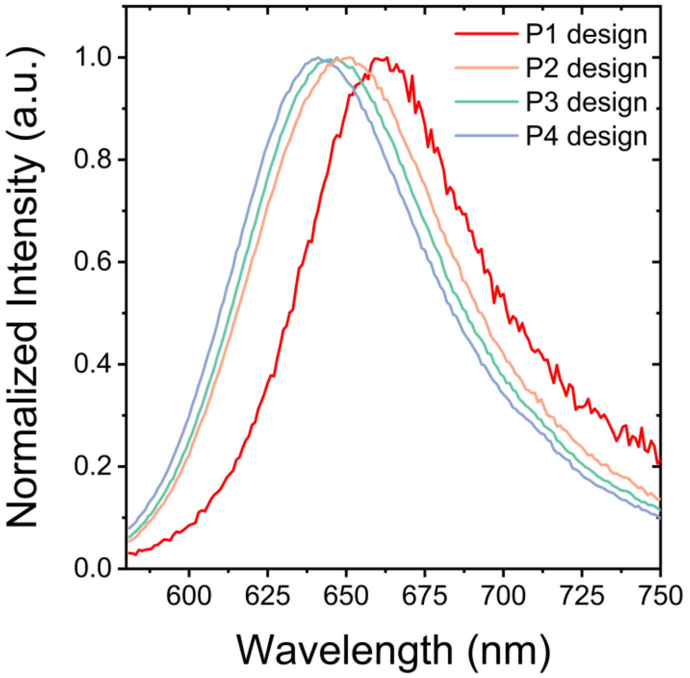

Figure S15: Normalized Nile Red fluorescence measurement of **P1, P2, P3, P4** in water (excitation wavelength 532 nm). $\lambda_{max,em}$ (**P1**) = 664 nm; $\lambda_{max,em}$ (**P2**) = 652 nm; $\lambda_{max,em}$ (**P3**) = 648 nm; $\lambda_{max,em}$ (**P4**) = 644 nm.

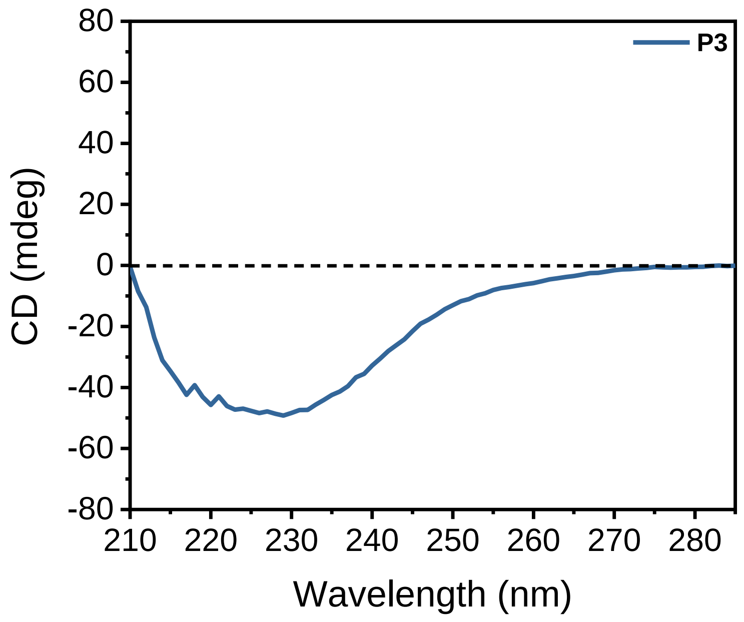

Figure S16: CD spectra of **P3** (c_P =0.35 mg mL^{-1}, T = 20 °C, optical path length l = 10 mm) in water.

4. Instruments

NR-sPAINT microscopy instrumentation. NR-sPAINT fluorescence imaging was performed using the same previously described inverted wide-field optical microscope configured with a grating to perform various spectrally-resolved single-molecule localization microscopy techniques.[3] Here, Nile Red excitation was directed off a dichroic mirror (ZET 532/10x) through an oil-immersion objective lens (Nikon Apo TIRF 100x Oil DIC N2) with a numerical aperture of 1.55 and a magnification of 100x to the coverslip. The sample was illuminated in total internal reflection fluorescence (TIRF), with a 50 mW, 532 nm continuous fiber-coupled wavelength source (FP1280764, Coherent OBIS). The fluorescence emission was collected through the same objective and passed through a longpass filter (ET542LP, Chroma Technology USA) and a notch filter (NF533-17, Thorlabs), before being expanded by a 1.5x relay lens. Finally, the emission was passed through a mechanical slit (VA100C, Thorlabs) and a transmission diffraction grating (70 grooves/mm, 25x25 mm-46-068, Edmund). By using a diffraction grating in front of the camera, the fluorescence signal is split into i) the 0th order diffraction representing the spatial domain and ii) the 1st order diffraction representing the spectral domain. The grating was selected considering that the detection of fluorescence emission is highly dependent on the photon budget that transmits at the spatial and spectral domain. It split the emission light 41% into the 0th order and 32% into the 1st order (based on the manufacturer for a 647 nm laser) and was mounted before an EMCCD camera (Andor DU-888 X-9414) with an electron multiplication gain of 250, exposure time of 50 ms and a pixel size of 90 nm. Then by adjusting the grating-camera distance (approximately 2 cm) and by calibrating the pixel to wavelength ratio, the NR-sPAINT spectral dispersion (SD) and spectral precision (σ) were calculated: 4.8 nm/pixel and 11 ± 2 nm, respectively, using a previously reported methodology (Figure S19).[4]

5. Sample preparation for NR-sPAINT measurements

Coverslip preparation for NR-sPAINT microscopy. Coverslips #1.5 (24×24 mm^2) were placed into an ultrasound bath with fresh methanol for 15 min. After they were dried with nitrogen, they undergo plasma treatment for 1 min (Openair FG 5001-Plasma Generator). For the sample preparation, the coverslip and glass slide (25×75 mm^2) were sandwiched by two strips of double-sided tape to form a capillary chamber with inner volume of ~30 μL.

SCPN immobilization for sPAINT microscopy. In order to prepare the glass surface, 30 μL of 0.1 mg/ml biotinylated bovine serum albumin dissolved in buffer A (10 mM TRIS-HCl, 50 nM NaCl, pH 8.0) was flown into the chamber and incubated for 1 hr. The unbound BSA was washed away by 200 μL of buffer A and subsequently with 200 μL of Milli-Q for buffer exchange. SCPNs of 4.2 μM, were mixed with 0.03 mg/ml streptavidin and shaken (300 rps) at room temperature for 45 min. The particles were flown into the chamber and incubated for 45 min. Then, they were washed with 100 μL of Milli-Q and 100 μL of PBS. Freshly prepared Nile Red in PBS was injected into the chamber before imaging. This was the imaging solution. All incubation steps were done in a humidity box to prevent drying, and the chambers were sealed at both ends.

Imaging with Nile Red. First, Phosphate-Buffered saline from ThermoFisher Scientific was freshly prepared in Milli-Q water and filtered (0.02 μm) and used for the preparation of the Nile Red solution for imaging. Nile Red stock solutions (250 μM) were prepared by dissolving Nile Red in high purity dimethyl sulfoxide (DMSO) and then diluted into the PBS buffer (pH 7.5). Finally, Nile Red was used at a final concentration of 5 nM unless otherwise specified.

6. Calibration of the instrument & spectral precision

Pixel to wavelength calibration. 100 nm Tetraspeck beads labelled with different dyes (ThermoFisher Scientific Massachusetts, US, T7279) were imaged using a 532 nm for 100 frames with exposure time of 90 ms. The raw data were used in RainbowSTORM which automatically extracted the pixel to wavelength relation within 550 nm and 750 nm spectra window.

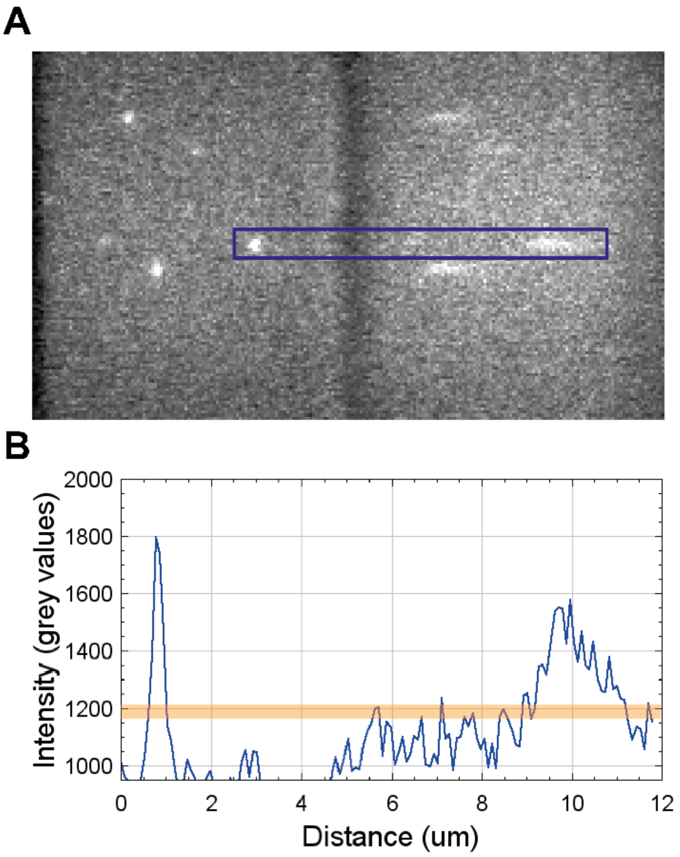

Figure S17: (A) Representative single frame of SCPN during NR-sPAINT imaging. (B) Respective raw spectra of this specific event in. The distance between the spatial localization and the bright stripe defines the actual wavelength of this specific binding event. The background threshold is depicted in orange.

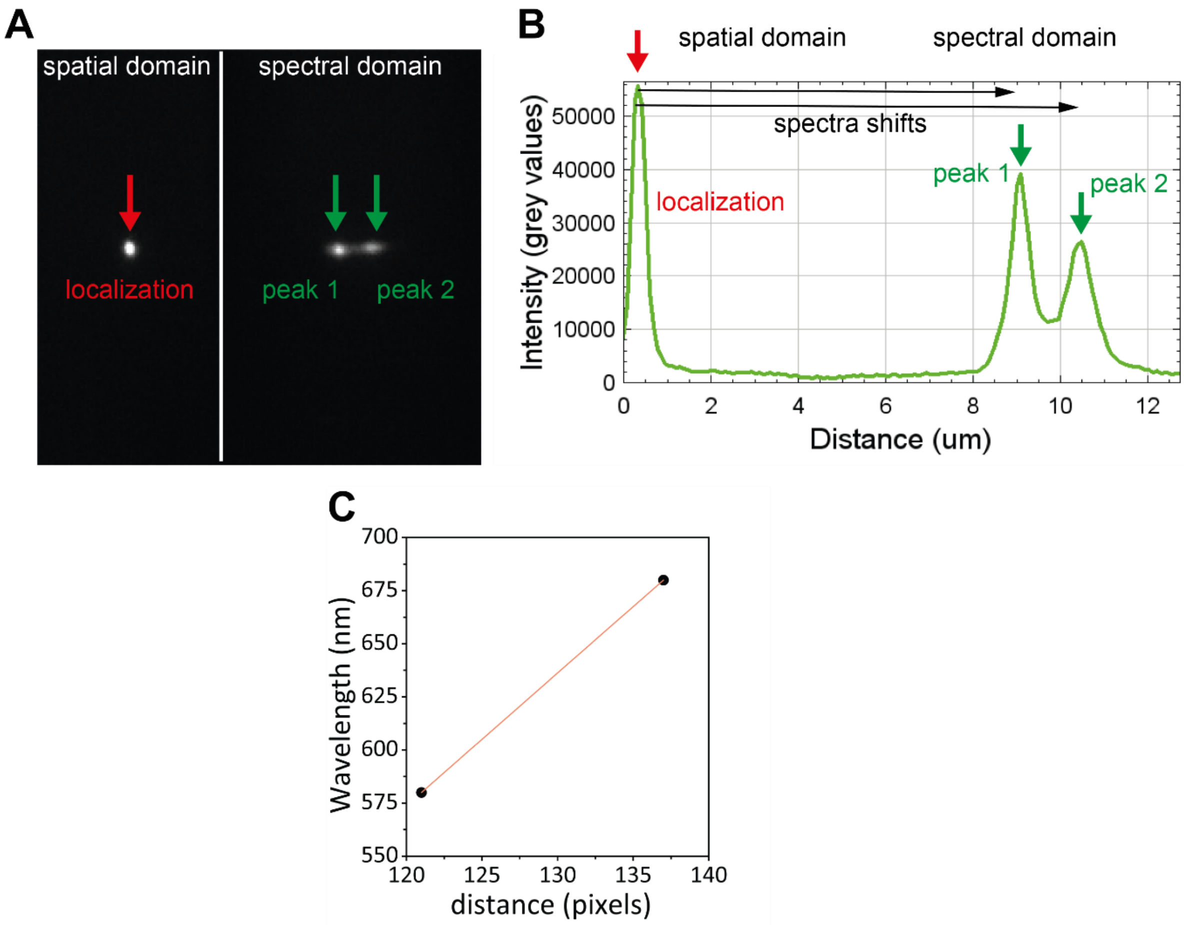

Figure S18: (A) Representative single frame of a Tetraspeck bead which was used for calibration because of their known emission peaks. After exciting the bead with a 532 nm laser source, a bright stripe is generated at the spectral domain. Since these beads are labelled with known multiple dyes, two discrete bright points are visualized on the stripe (two green arrows). These two points correspond to 580 nm and 680 nm emission peaks, respectively and can be used as reference. (B) The distance from the center of the localization to the center of each corresponding peak defines a specific wavelength value. (C) The two peaks and the distances can be used for the calibration (wavelength to pixel ratio) of the NR-sPAINT microscope.

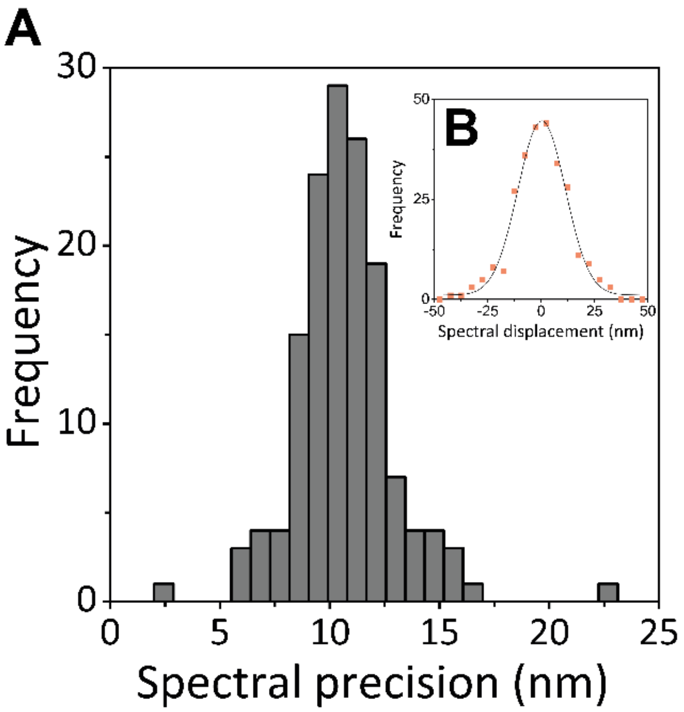

Figure S19: (A) Spectral precision histogram of NR-sPAINT microscope using 140 SCPNs was calculated using the previously reported supplementary methodology.[4] (B) First, the difference between the peak of each single-molecule from the mean polarity of the particle (spectral displacement) was calculated and binned for all the single-molecules on each SCPN. Then, all spectral displacements were binned in order to be fitted with a gaussian model and extract the single-particle spectral precision in A.

7. Controls

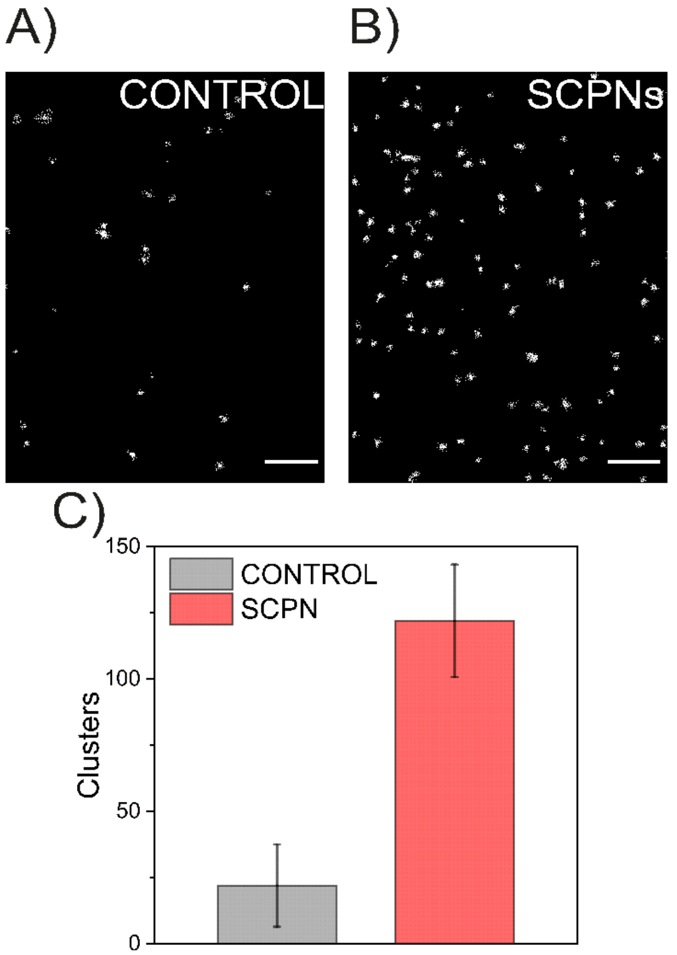

Figure S20: Clusters counted in the field of view. (A) Control coverslip (BSA + *Streptavidin) versus* (B) **P3**-based SCPN coverslip (BSA + Streptavidin + 0.5 nM SCPN). (C) Quantitative comparison from multiple regions of interest. Scale bar, 1 µm.

8. Single-molecule quantification

Binding quantification (spatial domain). First each localization in the spatial domain was detected using Thunderstorm plugin (ImageJ) which allowed for the extraction of the single-molecule localization coordinates (X, Y, T).[5] Next, a density filter is used in those images to remove most of the sparse non-specific localizations (minimum 5 in a 50 nm radius circle). In order to identify single-molecules in NR-sPAINT images we used a custom MATLAB algorithm. Firstly, localizations in spatial domain were merged into binding events (maximum frame gap of 3 frames in 40 nm distance with 5 minimum localizations per cluster). A mean-shift clustering algorithm is used to identify clusters in the spatial domain corresponding to specific events on the SCPN whilst ignoring the sparse non-specific ones (bandwidth of 40 nm and minimum 5 localizations per cluster). Mean-shift clustering is a non-parametric analysis that identifies local dense point spots of specific circular shape by shifting a window toward the density maximum inside that bandwidth. Clusters which have less than 15 events or are rather concentrated in a short period of time were filtered out. The mean dark (τ_d) and bright times were extracted for every valid cluster considering the temporal distance between consecutive events and the duration of the events respectively. Finally, by assuming that each isolated cluster corresponds to one SCPN target $(\mathbf{N = 1})$, Nile Red concentration fixed $(\mathbf{C_{NR} = 5\ nM})$ to allow for adequate events per SCPN, as well as a relative balanced SCPN concentration on coverslip (300 pM) to prevent aggregates and spectral overlap of the stripes, $\mathbf{k_{on}}$ value was calculated using the second-order association rate of the binding reaction (**equation 1**).[6]

$$\mathbf{k_{ON}} = \frac{\mathbf{1}}{\tau_d \times \mathbf{N} \times \mathbf{C_{NR}}} \tag{1}$$

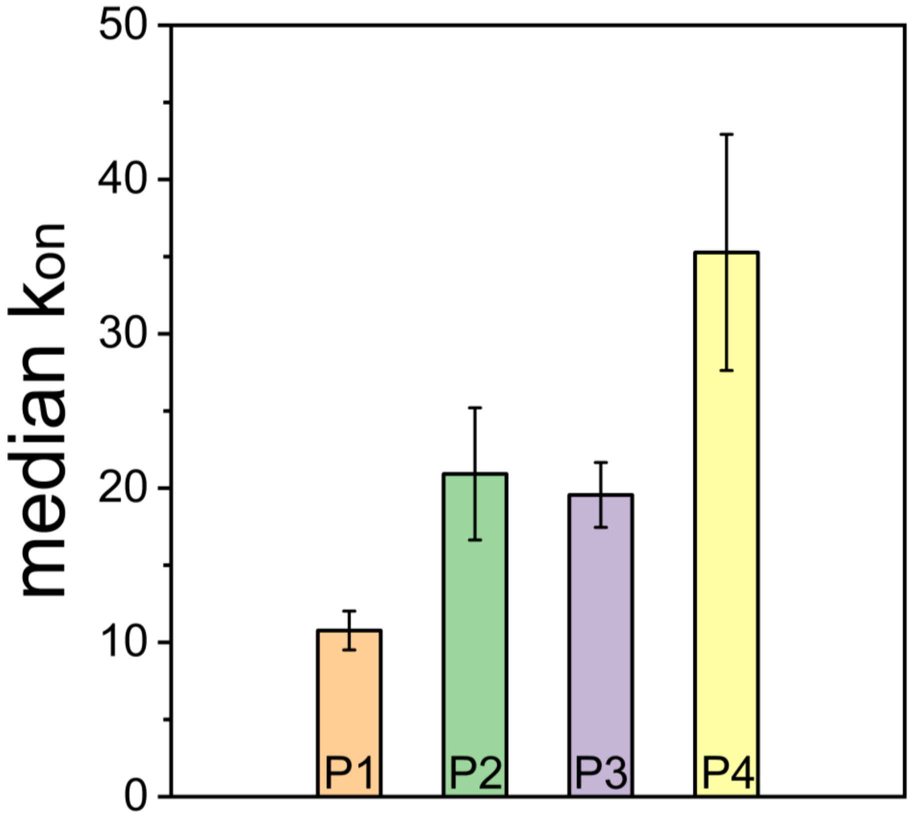

Figure S21: PAINT measurements on **P1-P2-P3-P4**. Each column contains independent experiments taken on different days. Each experiment contains different measured field of views (FOVs). For each experiment the median k_{on} values of the disperse distributions were evaluated. The increase of hydrophobic pendant groups on the polymer backbone increases a fraction of SCPNs which exhibit higher k_{on}. Imaging settings: shutter speed 50 ms, frames 20000, Nile Red concentration 5 nM. More than 200 hundred SCPNs were used for each design and compared.

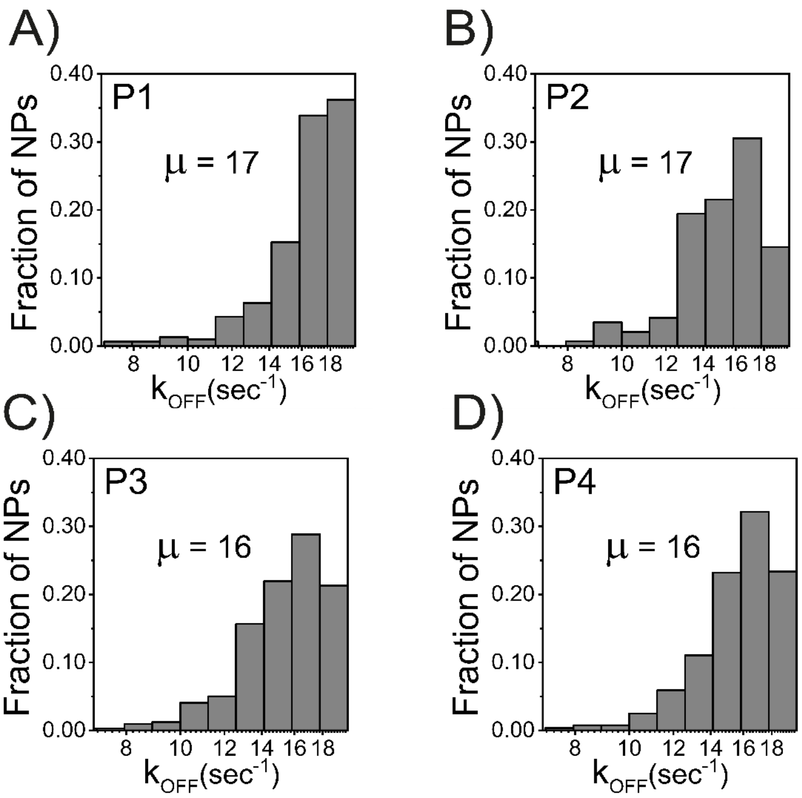

Figure S22: k_{off} histograms in **P1-P4** series (μ corresponds to the median of the population).

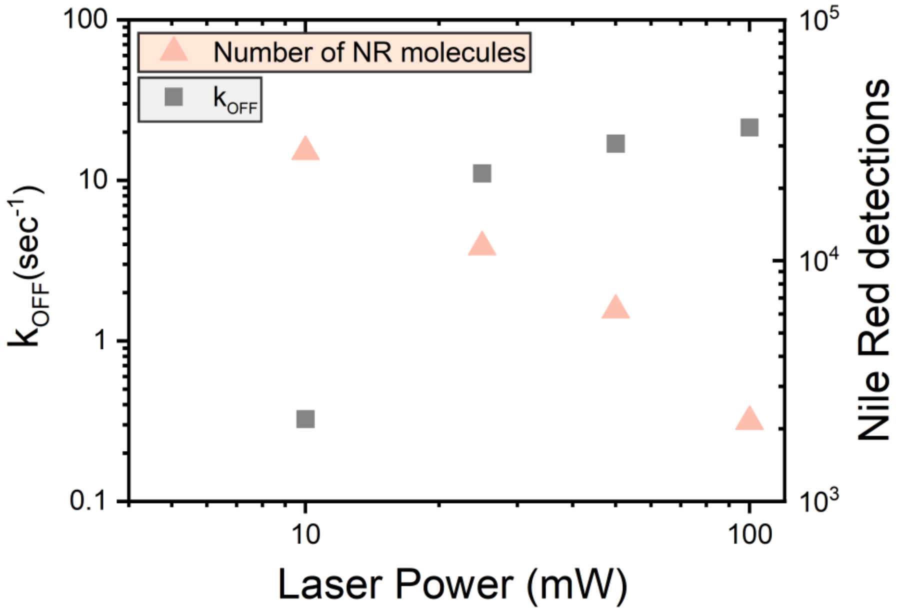

Figure S23: NR-PAINT measurements on a bare hydrophobic glass slide varying the power of the laser. Each grey point is one k_{off} measurement on the glass. Each red point represents the amount of molecules detected in the same area. Imaging settings: shutter speed 50 ms, frames 10000, Nile Red concentration 5 nM).

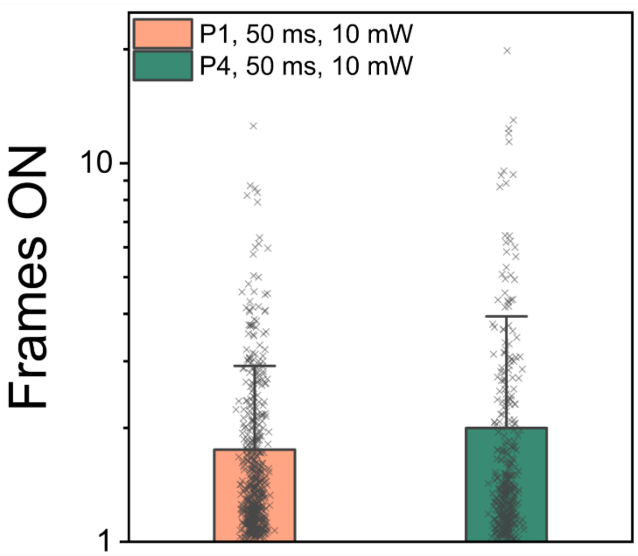

Figure S24: NR-PAINT measurements between **P1** and **P4** design. Laser power and exposure time were decreased in order to try capturing potential k_{off} differences between the two extreme samples. Every cross represents the average ON-time of all events on a single-particle level. In all cases, the events remain ON for a very short period (~1-2 frames). Imaging settings: shutter speed 50ms, frames 20000, Nile Red concentration 5 nM.

Polarity quantification (spectral domain). The corresponding stripes at the spectral domain were first analyzed using the set of coordinates which were extracted previously with the RainbowSTORM plugin (ImageJ)[7] in combination with the calibration curve which converts the spatial-spectra distance to actual wavelengths. Lastly all actual spectra of the events which belong to each SCPN were fitted using a gaussian peak fit function to extract the corresponded single-molecule wavelength values. The mean polarity per SCPN was extracted by calculating the mean of the events.

10. **References**

(1) ter Huurne, G. M.; de Windt, L. N. J.; Liu, Y.; Meijer, E. W.; Voets, I. K.; Palmans, A. R. A. Improving the Folding of Supramolecular Copolymers by Controlling the Assembly Pathway Complexity. *Macromolecules* **2017**, *50* (21), 8562–8569.

(2) Liu, Y.; Pauloehrl, Th.; Presolski, S. I.; Albertazzi, L.; Palmans, A. R. A.; Meijer, E. W. Modular Synthetic Platform for the Construction of Functional Single-Chain Polymeric

Nanoparticles: From Aqueous Catalysis to Photosensitization. *Journal of the American Chemical Society* **2015**, *137* (40), 13096–13105.

(3) Archontakis, E.; Woythe, L.; Hoof, B. van; Albertazzi, L. Mapping the Relationship between Total and Functional Antibodies Conjugated to Nanoparticles with Spectrally-Resolved Direct Stochastic Optical Reconstruction Microscopy (DSTORM). *Nanoscale Adv.* **2022**.

(4) Bongiovanni, M. N.; Godet, J.; Horrocks, M. H.; Tosatto, L.; Carr, A. R.; Wirthensohn, D. C.; Ranasinghe, R. T.; Lee, J.-E.; Ponjavic, A.; Fritz, J. V.; Dobson, C. M.; Klenerman, D.; Lee, S. F. Multi-Dimensional Super-Resolution Imaging Enables Surface Hydrophobicity Mapping. *Nat Commun* **2016**, *7* (1), 13544. .

(5) Ovesný, M.; Křížek, P.; Borkovec, J.; Švindrych, Z.; Hagen, G. M. ThunderSTORM: A Comprehensive ImageJ Plug-in for PALM and STORM Data Analysis and Super-Resolution Imaging. *Bioinformatics* **2014**, *30* (16), 2389–2390. .

(6) Jungmann, R.; Avendaño, M. S.; Dai, M.; Woehrstein, J. B.; Agasti, S. S.; Feiger, Z.; Rodal, A.; Yin, P. Quantitative Super-Resolution Imaging with QPAINT. *Nature Methods* **2016**, *13* (5), 439–442. .

(7) Davis, J. L.; Soetikno, B.; Song, K.-H.; Zhang, Y.; Sun, C.; Zhang, H. F. RainbowSTORM: An Open-Source ImageJ Plug-in for Spectroscopic Single-Molecule Localization Microscopy (SSMLM) Data Analysis and Image Reconstruction. *Bioinformatics* **2020**, *36* (19), 4972–4974.
